# Poor clinical outcome in metastatic melanoma is associated with a microRNA-modulated immunosuppressive tumor microenvironment

**DOI:** 10.1186/s12967-020-02235-w

**Published:** 2020-02-05

**Authors:** Natasha A. N. Jorge, Jéssica G. V. Cruz, Marco Antônio M. Pretti, Martín H. Bonamino, Patricia A. Possik, Mariana Boroni

**Affiliations:** 1 Bioinformatics and Computational Biology Lab, Division of Experimental and Translational Research, Brazilian National Cancer Institute, Rio de Janeiro, RJ 20231-050 Brazil; 2 Program of Immunology and Tumor Biology, Division of Experimental and Translational Research, Brazilian National Cancer Institute, Rio de Janeiro, RJ 20231-050 Brazil; 3 Vice Presidency of Research and Biological Collections, Fundação Oswaldo Cruz, Rio de Janeiro, RJ 21040-900 Brazil

**Keywords:** Metastatic melanoma, Tumor microenvironment, Immune evasion, miRNA, Tumor-TME crosstalk

## Abstract

**Background:**

Interaction between malignant cells and immune cells that reside within the tumor microenvironment (TME) modulate different aspects of tumor development and progression. Recent works showed the importance of miRNA-containing extracellular vesicles in this crosstalk.

**Methods:**

Interested in understanding the interplay between melanoma and immune-related TME cells, we characterized the TCGA’s metastatic melanoma samples according to their tumor microenvironment profiles, HLA-I neoepitopes, transcriptome profile and classified them into three groups. Moreover, we combined our results with melanoma single-cell gene expression and public miRNA data to better characterize the regulatory network of circulating miRNAs and their targets related to immune evasion and microenvironment response.

**Results:**

The group associated with a worse prognosis showed phenotypic characteristics that favor immune evasion, including a strong signature of suppressor cells and less stable neoantigen:HLA-I complexes. Conversely, the group with better prognosis was marked by enrichment in lymphocyte and MHC signatures. By analyzing publicly available melanoma single-cell RNA and microvesicle microRNAs sequencing data we identified circulating microRNAs potentially involved in the crosstalk between tumor and TME cells. Candidate miRNA/target gene pairs with previously reported roles in tumor progression and immune escape mechanisms were further investigated and demonstrated to impact patient’s overall survival not only in melanoma but across different tumor types.

**Conclusion:**

Our results underscore the impact of tumor-microenvironment interactions on disease outcomes and reveal potential non-invasive biomarkers of prognosis and treatment response.

## Background

Metastatic melanoma is a very aggressive and lethal disease. The high metastatic capacity and therapy resistance indicate a poor prognosis, such that the survival rate of advanced melanoma patients is approximately  one year and most patients succumb to the disease within three years of diagnosis [1]. The use of targeted- and immunotherapies is expected to increase patient survival, however, most melanoma patients continue to present poor prognosis due to therapy resistance, mainly after metastasis [1, 2]. Therefore, there is a need for studies dissecting melanoma biology, its pathogenesis, and relationship with the immune system that could aid the identification of new treatment strategies. Numerous studies have confirmed that tumor progression and recurrence are shaped by the tumor microenvironment (TME) [3, 4] besides the genetic changes inherent to the cancer cells. During melanomagenesis, both tumor cell proliferation and apoptosis are shaped by the activity of immune cells [5]. In this process called immunoediting [6], the features that suppress the cytotoxic immune infiltrate and promote tumor survival are positively selected. The understanding of these mechanisms has been crucial for treatment improvement and for the development of new immunotherapy strategies. Although immunotherapy approaches targeting CTLA-4 and PD-1 have been successful for melanoma and other cancer types [7], meaningful clinical responses have only been observed for a subset of patients. The observed variation in treatment efficacy has been related to heterogeneity in the composition of immune cells among individual tumors as well as the tumor mutation burden and expression of immune checkpoint molecules, but these factors alone cannot accurately predict a successful outcome of patients treated with immune checkpoint blockade [8, 9].

In this sense, many efforts have been made in order to characterize the composition of major immune cell subsets present in the TME. The T and B cell receptor (TCR and BCR) repertoire, the neo-antigenic immune targets [10–13] and, more recently, finer definitions of the frequency of immune cell subsets in tumors have revealed important molecular heterogeneities that are not intrinsic to the melanoma cells, but extend to the associated tumor components that shape the tumor microenvironment [14].

Besides characterizing the TME composition and the molecular alterations in tumors, it is crucial to understand how cancer cells and the surrounding neighbor cells are communicating to promote a favorable tumor growth niche. This crosstalk can occur indirectly, as for example through the release, by the tumor cells, of extracellular vesicles (EVs) containing oncogenic proteins, microRNAs (miRNA), messenger RNAs (mRNA) and DNA that are absorbed by the surrounding fibroblasts, immune, and endothelial cells altering their behavior in favor of tumor progression and metastasis [5], and vice versa. miRNAs from melanoma-derived exosomes have been implicated in the activation of cancer-associated fibroblasts (CAFs), in epithelial to mesenchymal transition, in neovascularization, and in the inhibition of the adjacent immune cell populations [15]. Some studies also suggest that circulating levels of certain miRNAs such as let-7a, mir-149, mir-211, and mir-191, are considered biomarkers of melanoma diagnosis and prognosis [16].

Although several mechanisms of immune evasion have been identified [17–19] and the effects of infiltrating immune cells on prognosis have been extensively reported for different tumor types [12, 13], the immune microenvironment diversity and the reciprocal interplay between melanoma and non-tumoral host cells shaping the disease progression and patient’s outcome is not fully understood. Therefore, the understanding of the specific contributions of each cell type to tumor growth can be a step toward developing new therapeutic strategies, predicting treatment resistance and avoiding selection of resistant populations. Because melanoma cells bear a peculiar immunogenic profile due to its higher mutational burden, melanoma tumors provide a suitable model to investigate the molecular crosstalk between cancer cells and cells of the immune system. Here, we undertook a large-scale, high-dimensional analysis of human metastatic melanoma samples, characterized the composition of the TME associated with important tumor features, and identified potential miRNAs from tumor cells that can modulate the immune system and vice versa. The identified miRNAs impact not only the TME and survival of melanoma patients but also of patients diagnosed with other very common and lethal tumor types such as breast, lung, ovary and esophageal tumors.

## Materials and methods

### Data download and sample selection

Gene expression data (HTSeq FPKM and HTSeq counts), pathological and clinical information from the “The Cancer Genome Atlas—TCGA” (http://cancergenome.nih.gov/) database portal were downloaded on 04/12/2018 using the R environment package TCGAbiolinks [20], using the identifiers: metastatic melanoma—SKML, lung adenocarcinoma—LUAD, breast—BRCA, esophageal carcinoma—ESCA, and ovarian cancer—OV. We also downloaded the RNA-Seq reads from melanoma tumors from the dbGaP website, under phs000178.v10.p8, and the annotated vcf files of three variant calling programs (MuSE, Varscan2, and SomaticSniper) for each analyzed sample using the SRA toolkit. From 173 metastatic samples from which the TME cell types could be predicted (“Materials and methods”), we removed samples that presented less than 50% of tumor cells or more than 50% of lymphocyte infiltration according to the available histopathological information, samples identified by histology as nevus and amelanotic melanoma, and samples annotated as “Primary Tumor” regarding the “submitted tumor location” field, totaling 164 samples (Additional file 1: Figure S1A). Details of all samples can be found in Additional file 1: Table S1. We also downloaded gene expression data from extracellular vesicles found in plasma samples of melanoma patients and healthy individuals [21]. The microarray pre-processed data (GEO accession: GSE100508) was downloaded using the GEOquery bioconductor package [22], normalized and log2 transformed. Mann–Whitney’s test was used to identify significant changes between groups (adjusted p < 0.05).

### Tumor microenvironment

The log transformed normalized RNA-Seq counts by DESeq2 [23] were used in CIBERSORT [10] with default parameters to infer the TME-associated immune cell types of each metastasis sample. The program is based on a deconvolution algorithm that infers relative frequencies of 22 immune cell types from its own gene signature. TME inferences with a p-value lower than 0.05 were kept for subsequent analysis. To confirm the CIBERSORT predictions, we used the same data in xCell [11], a software-based on gene set enrichment analysis to identify a variety of TME population signatures. Scores were filtered for p-value lower than 0.05 and the median for each cell population by group was calculated. Although focused on identifying TME composition, xCell and CIBERSORT use different mathematical approaches and gene signatures to identify TME cell types.

### Clusterization and group identification

Samples were grouped by non-supervised hierarchical clustering using the hclust function according to the immune cell type fractions predicted with CIBERSORT. Distance was measured as 1 − Pearson correlation coefficient. The closest samples were clustered using a height of 0.4 for the cutree function. Only groups of 20 or more components were considered. Bootstrap analysis was performed using the package “fpc: Flexible Procedures for Clustering” [24], yielding clusterwise Jaccard bootstrap means for each group. The “clusterboot” function was used with the following parameters: B = 10,000, bootmethod = “boot”, cluster method = disthclustCBI, k = 5, cut = “number”, method = “average”.

### Survival analysis

To identify miRNAs and mRNAs that could potentially influence the differences observed in the survival rates of patients grouped according to TME predictions, the normalized expression levels of each differentially expressed miRNAs and mRNAs were used to classify samples in groups. Survival rates of patients expressing lower levels of a specific gene than the average expression (“Low”) were compared to those with the expression greater than or equal to the average expression (“High”). Time intervals used for survival analysis were corrected to account only for metastatic disease, starting at the submission of the metastatic specimen and ending on the last follow up or death [25]. The R packages for survival analysis “survival” and “survminer” were used to obtain the Kaplan–Meier 5-year survival curves and the log-rank test was used to compare survival estimates across different groups. Hazard Ratio (HR) and 95% confidence intervals (CIs) were based on maximum likelihood estimates for each covariate using a Cox regression model.

### Differentially expressed genes and enriched pathways

The miRNA and total RNA sequencing raw counts were normalized and genes differentially expressed between groups were tested using DESeq2 [23]. Adjusted p-value (Benjamini–Hochberg) equal or lower than 0.05 for miRNA and 0.001 for total RNA were considered as significant. Unsupervised clustering was performed using 1 − Pearson correlation coefficient. The GSEA analysis was performed considering all DEGs on the WebGestalt online platform [26] using the REACTOME database to obtain relevant biological pathways with an FDR < 0.05.

### miRNA/mRNA pairs and interactions

Associations between differentially expressed miRNAs (DEM) and cell type signatures were evaluated by Pearson correlations matrix, using p ≤ 0.05 and abs(r) ≥ 0.4 as cut-offs. The miRNAs targets were obtained using the R environment package multmiR [27]. We considered as miRNA targets the genes detected in at least one of the databases available in the package and that were not annotated as “weak” in the “support_type” field by miRTarBase. The potential miRNA targets were further filtered to keep only those presenting a significant negative Pearson correlation (r ≤ − 0.4, p ≤ 0.05). Possible circulating miRNAs were also identified with the SpidermiR package [28], gathering information from the miRandola database [29]. miRNAs potentially derived from melanoma cells were identified by analyzing their expression in the melanoma cell lines data deposited in the CCLE database [30]. Analysis of publicly available single-cell RNASeq (SCRS) melanoma data [14] was used to identify the putative cell of origin of the identified targets, where genes with a median of expression greater than the second quartile observed when considering all genes expressed in this cell type were classified as potentially expressed by that cell type.

### Mutation analysis and neoepitope prediction

To ensure the accuracy of variant calling for downstream steps, we kept only single nucleotide variants called by VarScan2 and at least another variant caller (MuSE or SomaticSniper). The insertions and deletions were obtained only from the VarScan2 vcf file. To identify neoepitopes, we filtered the variants to keep only coding change mutations present in genes expressed in the analyzed samples (FPKM greater than the lower quartile when considering all genes of the same sample). HLA-I typing was performed with Optitype v.1.3.1 [31] and served as input to netMHCpan 4.0 predictions [32] together with the filtered vcf files. Peptides listed as strong binders (%Rank < 0.500) were called neoepitopes. The KD comparison across groups considered the median KD for all predicted neoepitopes per sample excluding outliers. Genes of the antigen processing and presentation pathway were obtained from the KEGG database [33] (Additional file 1: Table S2) and only mutations annotated by Variant Effect Predictor as with high or moderate impact and as with damaging/deleterious impact predicted by SIFT and Polyphen were considered. Mutational signature was performed using deconstructSigs package with hg38 [34].

### TCR and BCR repertoire

The T-cell receptor (TCR) and B-cell receptor (BCR) sequences were obtained using MiXCR 2.1.11 [35] with standard options, excluding out of frame sequences and premature stop codons. Sequences from which the immunoglobulin isotype was not identified were excluded. BCR/Ig representation was calculated multiplying each Ig isotype clone count by their respective read count (frequency).

## Results

### Clinical outcome of melanoma patients is impacted by the immune-related tumor microenvironment

**Fig. 1:**
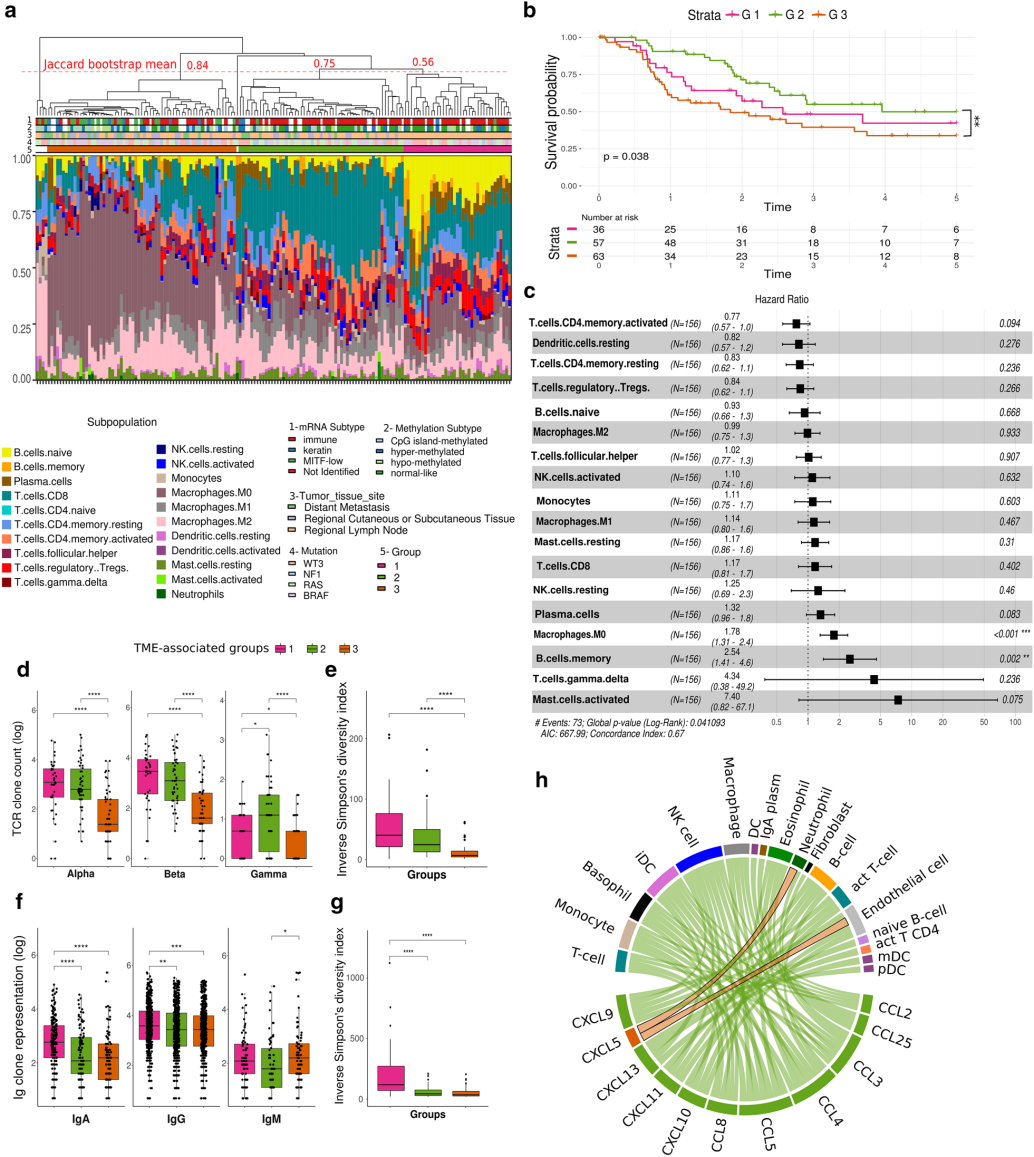
Metastatic melanomas can be distinguished according to their immune-related tumor microenvironment. **a** Hierarchical clustering of metastatic melanoma samples according to their predicted infiltrated immune cell populations. Samples were labeled according to their molecular subtypes, anatomical location and mutation status of the main driver genes. The dotted red line represents the cutoff for group assignment indicated in the label (G1—pink, G2—green, G3—orange) or no group (white) and values represent the mean clusterwise Jaccard bootstrap values calculated to confirm cluster stability, with 0.75 or higher pointing to stable clusters. **b** Kaplan–Meier curves for 5-year overall survival rates and Cox’s proportional hazards ratios of melanoma patients according to their TME-based classification. The log-rank test was used to analyze the difference in survival curves between the groups. Global p = 0.038. **p = 0.01 (Log-rank test for G2 versus G3). **c** Forest plot representing the survival hazard ratio of relative fractions, divided in quartiles, for each immune cell type using all samples of the three groups. **d** T-cell receptor (TCR) clone count (in log scale) per TCR chain. **e** Inverse Simpson diversity index of combined TCR chains. **f** B-cell receptor (BCR) representation (in log scale) considering immunoglobulins (Ig) isotypes. BCR representation was calculated by multiplying clonotype count by the correspondent read counts. **g** Inverse Simpson diversity index of combined BCR chains. MW test was used for mean comparisons: *p ≤ 0.05, **p ≤ 0.01, ***p ≤ 0.001, ****p ≤ 0.0001. **h** Circus plot showing differentially expressed chemokines between G2 and G3 linked to their respective target TME population. Chemokines upregulated in G3 are represented in orange and those upregulated in G2 are in green

In order to characterize human metastatic melanoma samples according to their associated immune phenotypes, we inferred the tumor microenvironment (TME)-associated immune cell populations [10] of 164 metastatic samples from The Cancer Genome Atlas (TCGA) database (Additional file 1: Table S1). This allowed the identification of three major groups containing at least 20 samples each: G1 (pink) with 37 samples, G2 (green) with 57 samples, and G3 (orange) with 65 samples (Fig. 1a). Bootstrapping showed that G2 and G3 were highly stable clusters, with a clusterwise Jaccard bootstrap mean of 0.56 for G1, 0.75 for G2, and 0.84 for G3. Five samples did not cluster within any of the groups and were excluded from the subsequent analysis (white labels, Fig. 1a).

According to the TME predictions, G1 samples are enriched in naïve, memory and plasma B cells, and depleted in resting natural killer (NK) cells; G2 samples are enriched in CD8+ T cells, monocytes, and macrophages M1; and G3 samples are enriched in M0 macrophages and depleted in plasma cells, CD8+ T cells, memory activated CD4+ T cells, follicular T helper cells, activated NK cells, monocytes, and resting dendritic cells (p ≤ 0.05, Mann–Whitney test—MW) (Fig. 1a and Additional file 1: Figure S1A). Regarding the histopathological characteristics, G1 samples present a higher percentage of tumor cells and, conversely, a small percentage of stromal cells when compared to G2 and G3 samples (Additional file 1: Figure S1B). Compared to G1 samples, samples in G3 have more necrotic cells and samples in G2 have more lymphocyte infiltration (Additional file 1: Figure S1B). Importantly, all samples selected for the analysis had > 50% of tumor cells.

The TCGA consortium has previously classified melanomas in four molecular subtypes based on their genomic profiles (BRAF, RAS, NF1, and triple wild-type), three transcriptional subclasses based on their gene expression signatures (Immune, Keratin, MITF-low) and four methylation clusters based on their methylation patterns (CpG island-methylated, hyper-methylated, hypo-methylated and normal-like) [36]. We did not find any association between the immune-related groups described here and the molecular subtypes (Fisher’s exact test p = 0.4) or the methylation profiles identified (Fisher’s exact test p = 0.12) (Fig. 1a and Additional file 1: Table S1) [36]. However, regarding the transcriptomic classification of melanomas, we found that G3 was enriched with metastatic melanoma samples classified into the MITF-low subclass whereas melanomas clustered into the Immune subclass were more present among G1 and G2 (Fisher’s exact test p = 2.51e−06).

To confirm the identified TME profiles of each group, we validated our findings using an alternative gene signature-based approach [11] (Additional file 1: Figure S1C). We performed non-supervised clustering of the TME populations using the median of the population signature predicted for each group. Five different B-cell types, CD4+ T naive cell type and non-immune cell types such as microvascular endothelial cells showed a strong association with G1. G2 showed a strong signature of all T cell subpopulations, such as CD8+, regulatory T (Treg), Th1 and Th2 cells along with macrophages and basophils. Finally, G3 showed a strong signature of non-immune cell types such as smooth muscle cells, pericytes, neurons, and mesenchymal stem cells. Although this alternative approach does not cover the M0 macrophage signature, the presence of a weaker signature of other immune cell types in G3 and the overlap between the immune cell populations predicted by the two different approaches present in G1 and G2 confirm our initial classification into three distinct immune-related groups.

To investigate whether different TME compositions correlate with clinical outcome, we next assessed the overall survival of melanoma patients grouped according to our TME-based classification (Fig. 1b). Patients clustered in G2 (green line) showed significantly better overall survival than G3 patients (orange line) (p = 0.01, log-rank test, Hazard Ratio (HR) = 0.49, confidence interval (CI) .95 = 0.28–0.85). G1 patients’ overall survival did not differ from the other two groups. To better account for the impact of specific immune cell types on survival, we calculated the Hazard Ratio (HR) of the relative fractions of each cell type population divided into quartiles (Fig. 1c). We identified poorer HR related to the presence of memory B cells (p = 0.002, log-rank test, HR = 2.54, CI.95 = 1.41–4.6) and M0 macrophages (p < 0.001, log-rank test, HR = 1.78, CI.95 = 1.31–2.4), which are both immune cell types highly represented in G1 and G3, respectively. In addition, we analyzed the correlation among the different predicted immune cell types in all samples combined (Additional file 1: Figure S2A). This revealed several connections between cells that could potentially impact tumor growth and help explain the observed prognosis. Examples include a positive correlation between Treg cells and memory B cells or M0 macrophages (r = 0.47 and r = 0.56, respectively, p ≤ 0.05, Pearson correlation test—PCT) and a strong negative correlation between CD8+ T cells and M0 macrophages (r = − 0.41, p ≤ 0.05, PCT). Most cell types were positively correlated, of which the correlation between neutrophils and activated mast cells was the highest observed (r = 0.65, p ≤ 0.05, PCT), followed by CD4+ memory resting T cells and M2 macrophages (r = 0.62, p ≤ 0.05, PCT), and CD8+ T cells and CD4+ memory activated T cells (r = 0.61, p ≤ 0.05, PCT). Since we observed a significant difference between the overall survival of G2 and G3 patients, we next investigated which immune cell types better discriminate these groups by calculating the point-biserial correlation coefficient (Additional file 1: Figure S2B). We observed that monocytes were better correlated to G2 (r = 0.29, p ≤ 0.05, PCT), and M0 macrophages to G3 samples (r = − 0.27, p ≤ 0.05, PCT). Also, a strong and significant negative correlation between these two cell populations was observed (r = − 0.59, p ≤ 0.05, PCT).

Finally, we evaluated the frequency and diversity of T and B-cell receptors (TCR and BCR, respectively) as they correspond, at least in part, to the T and B lymphocyte populations observed. G1 and G2 presented higher numbers of unique alpha/beta chains clonotypes in the TCR repertoire (medians of 28.5 and 17, respectively) in comparison to G3 (median of 4 and p ≤ 0.0001 in both comparisons using MW test), but no significant difference was found between G1 and G2 (Fig. 1d). G2 also presented a greater variety of gamma chain clonotypes (median of 2) when compared to either G1 (median of 1; p = 0.011) or G3 (median of 0; p ≤ 4.9e10−6). The lymphocyte repertoire diversity, assessed by the inverse Simpson diversity index, was higher in G1 and G2 (p ≤ 9e10−8 for G1 and G2) compared to G3 (Fig. 1e). Higher TCR diversity in G1 and G2 supports the notion that more diverse antigen collections are being recognized and contribute to explain the better outcome of G2 patients. We also analyzed the BCR repertoire and found a higher immunoglobulin A (IgA) representation in G1 (median of 15 clones per sample; p ≤ 7.1e10−6) compared to G2 and G3 (median of 7 and 8, respectively) (Fig. 1f). The same was also true for IgG clonotypes which were more represented in G1 (median of 35) in comparison to G2 (p = 0.002, median of 30) and G3 (p = 0.0008, median of 30). G3 presented higher IgM representation (median of 8) in comparison to G2 (median of 5, p = 0.027). The BCR diversity was also higher in G1 compared to G2 and G3 (p ≤ 5.3e10−8) (Fig. 1g). In accordance with the variety of lymphocyte subpopulations, the expression of chemokines that recruit several of the G2-enriched immune cell populations was augmented in this group when compared to G3 (adjusted p-value ≤ 0.001), except for *CCL5*, which acts on neutrophils and endothelial cells (Fig. 1h). This lack of chemokine expression in G3 can explain, at least in part, the paucity of CD8+ T lymphocytes in these samples, a characteristic that underpins malignant development.

### Phenotypic characteristics that favor immune evasion co-occur in melanomas with worse prognosis

Besides avoiding the recruitment of inflammatory cells and lymphocytes to the TME, tumor cells can develop strategies to become “invisible” to the immune system or to inhibit effector responses. These strategies, based on distinct mechanisms of immune evasion, may have a significant impact on clinical outcome of cancer patients. Therefore, we characterized different aspects of known immune evasion mechanisms in the TME-grouped melanoma samples.

**Fig. 2:**
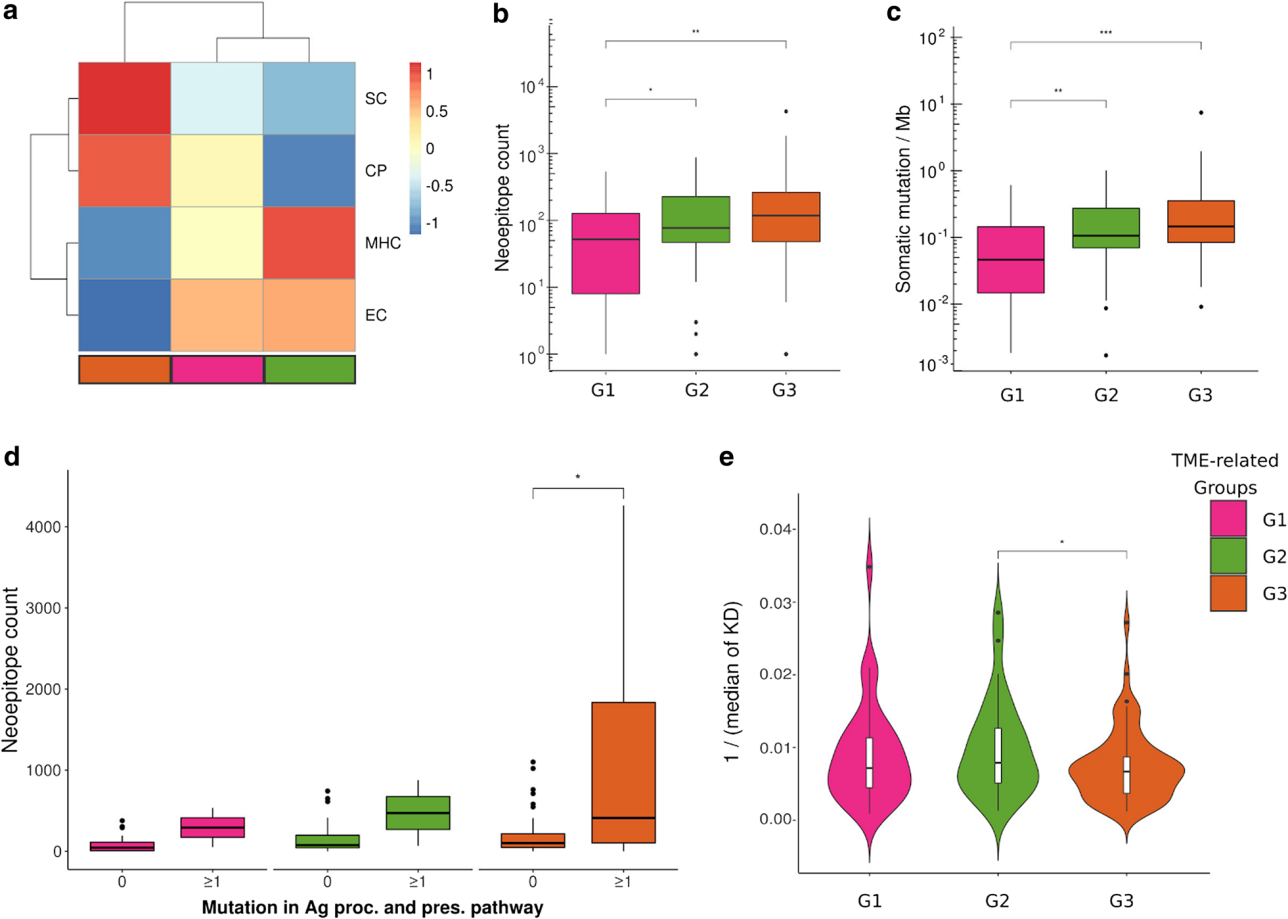
The composition of the tumor microenvironment reflects specific immunogenic features. **a** Immunophenoscore signatures of the TME-associated groups. Median values per group of signatures of suppressor cells (SC), checkpoint molecules (CP), MHC molecules and effector cells (EC) were Z-score transformed and compared. **b** Neoepitope counts (in log scale) predicted for each patient’s HLA-I allele. **c** Log values of somatic mutation burden per genome megabase (Mb). Only single nucleotide variants called by VarScan2 and at least one additional tool (MuSE or SomaticSniper) were considered. **d** Number of neoepitopes per group considering samples with/without deleterious mutations in genes belonging to the antigen processing and presentation pathway. **e** HLA-I binding affinity distribution of predicted neoepitopes among groups. For each sample, we used the median HLA-I binding affinity. Statistical analysis in **b**, **c** and **e** were performed by MW test: *p ≤ 0.05, **p ≤ 0.01, ***p ≤ 0.001. KD—dissociation constant for HLA-I binding in nM

First, we identified signatures of effector cells (EC), suppressor cells (SC), checkpoint molecules (CP) and major histocompatibility complex (MHC) expression in the three groups, which were previously shown to predict response to immune checkpoint blockade [19]. Weaker signatures of SC and CP were detected in G3 whereas a stronger signature of EC was observed in G1 and G2 (Fig. 2a). These results reinforce that ongoing effector responses are more likely to occur in G1 and G2 and that suppressor mechanisms are of great importance in G3 and could favor immune evasion.

Tumors with a higher mutation burden tend to generate more neoantigens and, thus, be more immunogenic [37]. We observed significantly higher numbers of somatic mutations per megabase (Mb) in G2 and G3 compared to G1 (p = 0.0069 and 0.00013, respectively, MW test, Fig. 2b). The median of exonic mutations per Mb was 60 for G1, 130 for G2, and 187 for G3 (Additional file 1: Figure S3A) alike the intronic mutations (Additional file 1: Figure S3B). Similarly, G2 and G3 samples presented more neoepitopes compared to G1, of which the median was 51 for G1, 77 for G2 and 120 for G3 (p = 0.026 and p = 0.0037, respectively; MW test, Fig. 2c). Despite observing no difference in neoepitope and mutation burden between G2 and G3, and based on the lower T and B cell diversity observed in G3 (Fig. 1e, g), we searched for features that could impact neoepitope processing and presentation and thus diminish the recognition of a tumor cell. First, we searched for high impact mutations in genes belonging to the antigen processing and presentation pathway (Additional file 1: Table S2) and identified an increase in neoepitope burden according to the number of genes mutated in the pathway in a given sample (Fig. 2d and Additional file 1: Table S3). Two samples from G1 and two from G2 had one mutated gene on this pathway while nine samples from G3 presented at least one gene mutated. Within G3, samples bearing mutations on this pathway had increased neoepitope burden when compared to the non-mutated samples (p = 0.022, MW test). No statistical test could be performed for G1 and G2 samples due to the small number of events.

Another feature that impacts neoepitopes presentation is the predicted affinity between them and the Human Leukocyte Antigen I (HLA-I) alleles so that higher dissociation constants (KD) account for lower stability of the neoepitope:HLA-I complex and therefore a less efficient presentation. The median of neoepitope’s KD was calculated for each sample and compared among groups (Fig. 2e). Neoepitopes’s KD were higher in G3 compared to G2 (median of 149.5 nM and 126.9 nM, respectively; p = 0.042, MW test), supporting the idea that the immune evasion phenotype in G3 samples may also involve selecting less stable neoepitope:HLA-I complexes.

Mutational signatures are consequences of different etiological agents that, in the process of mutational carcinogenesis, favor some specific DNA transversions and transitions [34] and these mutational signatures have been related to patient’s survival [38]. Therefore, we asked if the distinct mutational signatures were differentially represented across the immune-related groups. Two main mutational signatures were identified across the groups: signature 7 (green) and 1A (pink), both corresponding to C>T transitions (Additional file 1: Figure S3C). Signature 7 is associated to ultraviolet radiation and previously described as related to melanoma, while signature 1A is broadly present among tumors [34]. We observed a similar mutational signature profile across the immune-related groups, suggesting that they can not be distinguished by genomic signatures.

### Differential miRNA/mRNA expression profiles dictate the clinical outcome of melanoma patients

In order to better understand the complex gene regulatory network related to the differences observed in patients’ survival and the immune evasion profiles of tumors, we compared the gene expression profiles of G2 and G3 samples. We identified 1783 differentially expressed genes (DEGs) (adjusted p ≤ 0.001) (Additional file 1: Figure S4A, B) and 93 differentially expressed miRNAs (DEM) (adjusted p ≤ 0.05) (Additional file 1: Figure S4C, D), of which 641 DEG and 34 DEM were up- and 1142 DEG and 59 DEM were downregulated in G3 (worse prognosis). The top 20 DEGs comprise four upregulated (*AL035610.1*, *ST8SIA5*, *NRXN1* and *FAM131B*) and 16 downregulated (*IFNG*, *TMEM155*, *RP11-109E24.1*, *CD8A*, *KLRK1*, *AC104820.2*, *FASLG*, *CCL4*, *GZMA*, *RP11-1094M14.8*, *AKAP5*, *RP11-1094M14.5*, *CLEC2D*, *TRGC2*, *CTC-303L1.1* and *JAKMIP1*) genes (Additional file 1: Table S4), while within the top 20 DEMs, 12 were upregulated (mir-206, mir-203a, mir-183, mir-205, mir-6892, mir-675, mir-887, mir-200c, mir-375, mir-1-1, mir-1-2, mir-130b) and 8 were downregulated (mir-142, mir-7702, mir-342, mir-4494, mir-155, mir-4491, mir-150, mir-6842) (Additional file 1: Table S5).

**Fig. 3:**
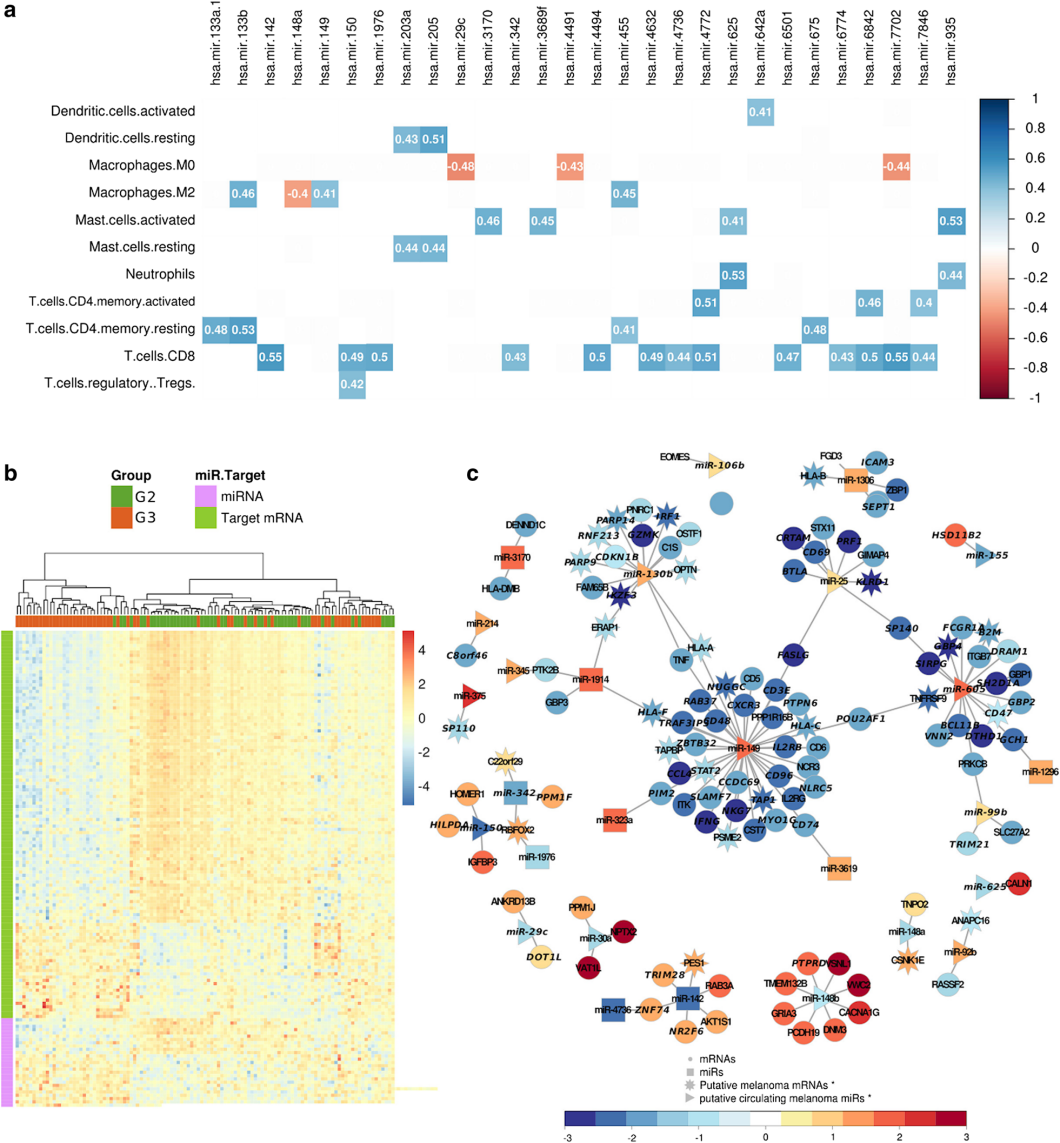
Differentially expressed miRNA/mRNA (MTG) pairs impact the overall survival of melanoma patients. **a** Pearson correlation matrix between immune cell types and differentially expressed miRNAs (DEM). Only correlation with abs(r) ≥ 0.4 and p-value ≤ 0.05 are shown. The immune cell types enriched in G2 and the miRNAs upregulated in G2 are highlighted in green and those in G3 in orange. **b** Heat map showing the expression pattern of miRNAs and target genes among samples, which were hierarchical clustered using distance as 1 − Pearson correlation coefficient. Data represent RNA-seq normalized pseudocounts in log2 scale, z-score transformed by rows. **c** Network highlighting the interactions between MTG pairs. The putative circulating miRNAs reported by miRandola are identified as triangles and their targets as stars. Gene expression in melanoma cells was accessed in the CCLE database. Node colors represent the gene expression levels in G3 related to G2. All genes in the network labeled in italic have an impact on patients’ survival

Gene Set Enrichment analysis (GSEA) revealed that the pathways enriched in G3 were involved in epithelial cell processes such as Keratinization and Extracellular Matrix Organization (False Discovery Rate (FDR) < 0.001, test = Permutation test for both) (Additional file 1: Figure S4E). The enriched pathways in G2 participate in immune response processes such as Downstream TCR signaling (FDR = 0.002, Permutation test), Interferon Signaling (FDR < 0.001, Permutation test), and Adaptive Immune System (FDR < 0.001, Permutation test). To analyze the potential impact of miRNA expression on the presence of each predicted cell type, analysis of the correlation of the DEMs and the predicted immune cell types was performed. A significantly negative correlation was found between miRNAs downregulated in G3 and M0 and M2 macrophages, including mir-148a: macrophage M2 (r = − 0.4, p = 8.81e−06, PCT) and mir-29c: macrophage M0 correlations (r = − 0.48, p = 7.68e−08, PCT) (Fig. 3a). Moreover, CD8+ T cell type, enriched in G2, was positively correlated to mir-142 and mir-7702 (r = 0.55 for both, p = 2.14e−10 and p = 1.93e−10, respectively, PCT) as well as to other 11 miRNAs. Dendritic resting cell type, decreased in G3, also correlated to mir-203a (r = 0.43, p = 1.57e−06, PCT) and mir-205 (r = 0.51, p = 7.16e−09, PCT).

In order to illustrate the relationship between miRNAs and the differences in gene expression in G2 versus G3, we determined miRNA-target gene (MTG) pairs. We identified 1111 DEGs predicted as targets of the DEMs (Fig. 3b), with 139 MTG pairs being negatively correlated, suggesting differences in the canonical regulation of mRNAs by miRNAs between the groups. We then sought to identify the interactions between melanoma and TME cells that were most likely to occur. For that, we used a manually curated list of mRNAs and miRNAs putatively expressed by melanoma cells retrieved from the Cancer Cell Line Encyclopedia (CCLE) and a publicly available list of circulating miRNA [29] and integrated this information into a gene expression network that shows the expression pattern of each gene in G3 relative to G2 (Fig. 3c). A hundred and thirteen MTG pairs (81%) involved putative circulating miRNA produced by melanoma cells suggesting that the DEMs are regulating the gene expression both at the intra- and intercellular levels. We also evaluated the impact of the expression of each MTG pair on patients’ outcome. In total, we found 74 MTG pairs with a significant impact on overall survival (p ≤ 0.05, log-rank test, Additional file 1: Figure S5). Of those, 12 pairs (mir-1296/*GCH1*, mir-1306/*ZBP1*, mir-1306/*ICAM3*, mir-1306/*SEPT1*, mir-142/*NR2F6*, mir-142/*TRIM28*, mir-142/*ZNF74*, mir-1914/*HLA-F*, mir-323a/*PIM2*, mir-3619/*CD74*, mir-4736/*ZNF74* and mir-342/*PPM1F*) did not involve potentially circulating miRNAs.

### miRNA-based intercellular communication in the TME impacts patient’s survival

Analysis of bulk tumor samples allowed us to identify potential miRNA-target genes but not to predict which cells within the tumor were expressing the miRNA or the target gene. To refine these analyzes, we took advantage of two publicly available datasets. First, we analyzed the candidate miRNA-target gene expression at a cellular level using Single Cell RNA-Seq (SCRS) data from melanoma tumors [14]. Second, we used a dataset consisting of miRNA expression profiles in extracellular vesicles isolated from the plasma of metastatic melanoma patients (hereafter referred to as bearer patients), healthy individuals, and R0-operated patients whose melanomas were surgically removed (with clear margin). The latter was subdivided into high relapse-risk and low relapse-risk based on tumor staging [21]. When combining these two datasets, we identified circulating miRNAs that are potentially expressed by TME cells and whose targets are mainly expressed in tumor cells, as well as miRNA potentially expressed by tumor cells that may affect gene expression in cells from the microenvironment. We further investigated the impact of these miRNAs in overall survival across different types of malignancy (Additional file 2). We describe below some examples to depict the potential role of circulating miRNAs in the interplay between the TME and malignant cells.

### Circulating miRNAs potentially suppressing tumor growth

**Fig. 4:**
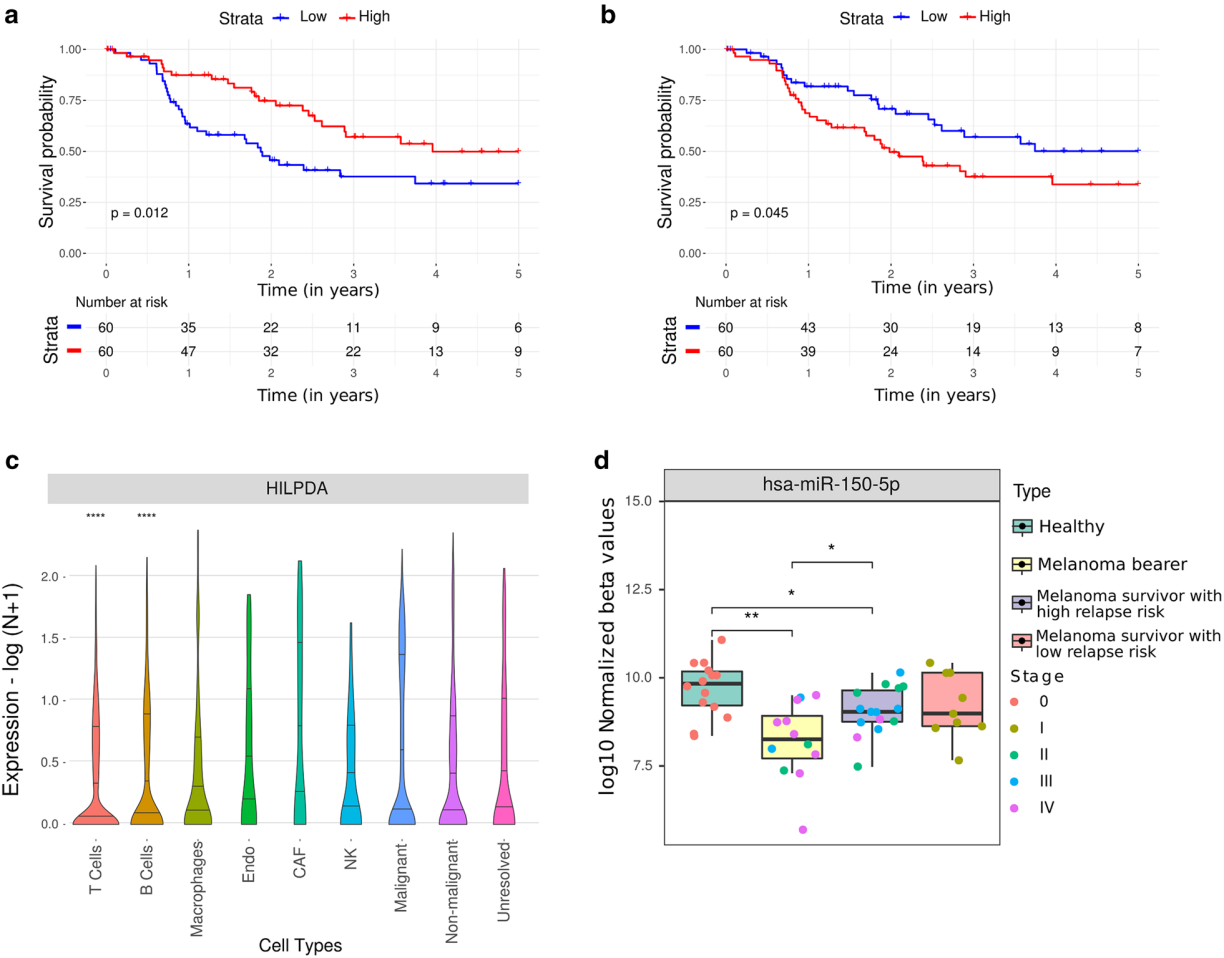
Putative TME-derived circulating miRNA associated with good prognosis in metastatic melanoma. Kaplan–Meier curves for 5-year overall survival rate of melanoma patients from G2 and G3 according to High (≥ average expression) or Low (< average expression) expression of **a** mir-150 (p = 0.012, log-rank test) and **b***HILPDA* (p = 0.045, log-rank test). **c** Violin plot showing the expression of *HILPDA* by cell type based on melanoma SCRS data. The malignant cell type was used as reference for MW test. **d** Boxplots of miR-150-5p expression levels (log 10 normalized beta values) in extracellular vesicles extracted from plasma samples. MW test was used to compare pairwise means from all groups. **p ≤ 0.01, ***p ≤ 0.001 and ****p ≤ 0.0001

One of the strongest MTG pairs identified in our analysis with a significant impact on patients’ prognosis was mir-150/*HILPDA* (Fig. 4a, b). The putative circulating mir-150 was found to be downregulated in G3. Consistently, its potential target, the Hypoxia-inducible lipid droplet-associated (*HILPDA*) gene, which encodes a protein related to intracellular lipid accumulation, was found to be upregulated in the same group (Additional file 2). analysis of SCRS data revealed that HILPDA was mainly expressed in malignant cells (Fig. 4c). The average expression of the mature form of hsa-miR-150-5p was lower in the extracellular vesicles from bearer patients (Log Fold Change (LFC): − 1.48, p = 0.001, MW test) when compared to healthy individuals (Fig. 4d). Interestingly, comparison of bearer and high-risk patients demonstrated a gain in hsa-miR-150-5p expression after surgical removal of the melanoma (LFC: 0.62, p = 0.03, MW test), although expression was still lower than in healthy individuals (LFC: − 0.62, p = 0.03). Higher levels of mir-150 were also related to better survival in lung adenocarcinoma and ovarian cancer (p = 0.011, log-rank test, HR = 0.67, CI.95 = 0.49–0.91, and p = 0.021, log-rank test, HR = 0.72, CI.95 = 0.53–0.95 respectively. Additional file 2). Altogether, these findings suggest that the release of hsa-miR-150-5p in EVs can be involved in the crosstalk between immune and tumor cells associated with suppression of tumor growth.

With similar characteristics, mir-342 was found to be downregulated in the group with worse prognosis, and its lower expression was associated with a worse overall survival in melanoma (p = 0.002, log-rank test, HR = 0.43, CI.95 = 0.25–0.75, Additional file 1: Figure S6) and breast carcinoma (p = 9.9e−04, log-rank test, HR = 0.51, CI.95 = 0.33–0.76, Additional file 2). Consistently, its target, the protein-coding gene *PPM1F*, of which higher expression was associated with a worse outcome (p = 0.033, log-rank test, HR = 1.77, CI.95 = 0.25–0.75, Additional file 1: Figure S5), was found to be upregulated in G3 (Additional file 2) and expressed in several cell types according to the SCRS analysis, including malignant cells (Additional file 1: Figure S7). Although not described as circulating miRNA in our initial analysis, hsa-miR-342-3p was downregulated in EVs from bearer patients when compared to healthy individuals (LFC = − 0.80, p = 0.001, MW test). Similar to what was observed for hsa-miR-150-5p, an increase in the expression of hsa-miR-342-3p was detected in the plasma of both high- and low-risk melanoma survivors when compared to metastatic melanoma patients (LFC = 0.61 and p = 0.004, LFC = 0.53 and p = 0.03, respectively, MW test, Additional file 1: Figure S8).

### Circulating miRNAs potentially favoring tumor growth

We also identified miRNAs whose high expression were associated with worse outcome across at least three different tumor types, such as the putative circulating mir-130b, which was up-regulated in the group with worse prognosis (LFC = 0.65, padj = 1.75e−03). Seven out of 15 mir-130b targets identified among the DEGs were mainly expressed in non-malignant cells, such as T and B cells. (Additional file 2). Our independent analysis on the circulating miRNA dataset showed that average expression of miR-130b-3p was higher in the plasma EVs of high-risk patients when compared to plasma samples from healthy individuals (LFC = 1.69, p = 0.03, MW test, Additional file 1: Figure S8) also indicating the putative tumor-promoting role of this miRNA.

**Fig. 5:**
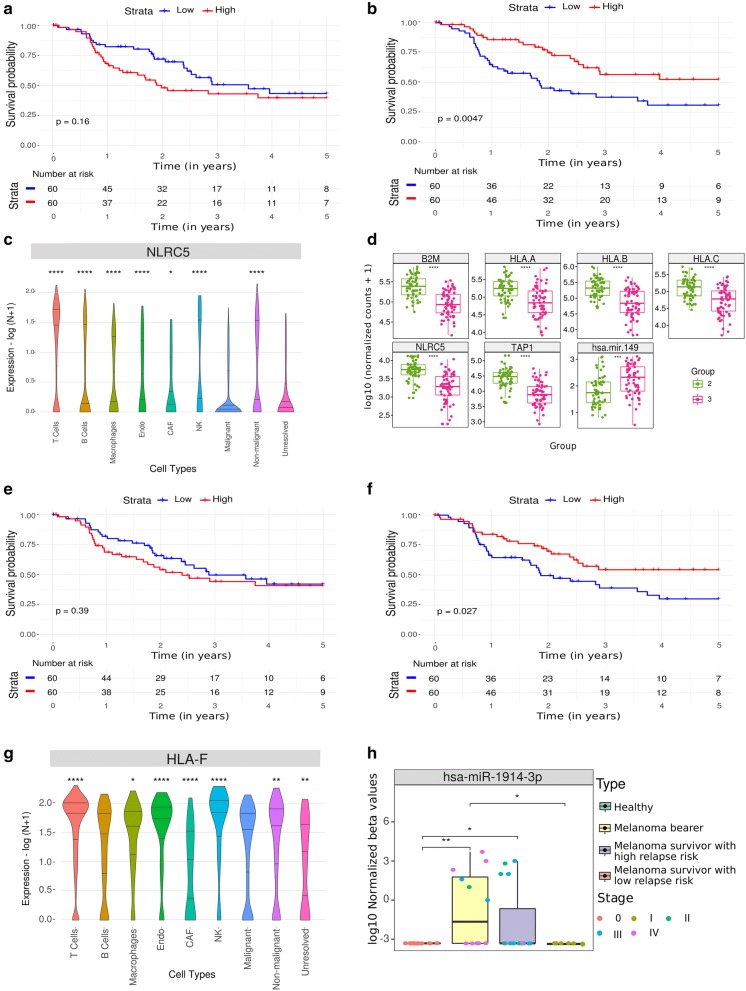
Putative tumor-derived circulating miRNA that modulates the TME. Kaplan–Meier curves for 5-year overall survival rate of melanoma patients from G2 and G3 according to High (≥ average expression) or Low (< average expression) expression of **a** mir-149 (p = 0.16, log-rank test) and **b***NLRC5* (p = 0.0047, log-rank test). **c** Violin plot showing the expression of *NLRC5* by cell type based on melanoma SCRS data. Asterisks inform on Mann–Whitney p values using the malignant cell type as reference. **d** Boxplots of the expression levels of mir-149, *NLRC5* and genes involved in the antigen presentation pathway (*TAP1*, *B2M*, *HLA*-*A/B/C*) on G2 (green) and G3 (pink). Asterisks inform on Mann–Whitney test p values. *p ≤ 0.05, **p ≤ 0.01, ***p ≤ 0.001, ****p ≤ 0.0001. **e** Kaplan–Meier curves for 5-year overall survival rate of melanoma patients from G2 and G3 according to High (≥ average expression) or Low (< average expression) expression of a mir-1914 (p = 0.39, log-rank test) and **f***HLA-F* (p = 0.027, log-rank test). **g** Violin plot showing the expression of *HLA-F* by cell type based on melanoma SCRS data. The malignant cell type was used as reference for MW test. **h** Boxplots of miR-1914 expression levels (log 10 normalized beta values) in extracellular vesicles extracted from plasma samples. MW test was used to compare pairwise means from all groups. **p ≤ 0.01, ***p ≤ 0.001 and ****p ≤ 0.0001

The putative circulating mir-149, which potentially regulates 39 targets of the 139 MTGs (Fig. 3c and Additional file 2), was upregulated in G3. Although the expression levels of mir-149 was not significantly associated with survival (Fig. 5a), all of its targets were downregulated in G3 and their low expression levels were associated with poor overall survival (Fig. 5b and Additional file 1: Figure S5). According to the melanoma SCRS data, all mir-149 targets identified were expressed in lymphocytes, some of which were exclusively expressed in this cell type and include *CD96*, *CD48*, *SLAMF7*, *FASLG*, *NUGGC* (Additional file 1: Figure S7) and *NLRC5* (Fig. 5c). Interestingly, *NLRC5* encodes a transcription coactivator of genes involved in HLA class I presentation, such as *TAP1*, *B2M* and *HLA-A/B/C*, all of which were downregulated in G3 (Fig. 5d), suggesting an immune evasion phenotype in G3 regulated by mir-149. It is worth to note that, among these genes, only *HLA-A/B* could not be associated with poor prognosis (Additional file 1: Figure S5). Unfortunately, the restricted expression of hsa-miR-149-3p in only two bearer patients combined with the absence of other mature forms of miR-149 in the microarray chip prevented us from assessing the differential expression of this miRNA in the EV dataset.

Another candidate MTG pair with a potential impact in the melanoma-TME crosstalk identified by our analysis was mir-1914/*HLA-F* (Fig. 5e, f). mir-1914 was upregulated in G3 (LFC = 1.12, padj = 1.17e−02) and was found to regulate four putative targets (Additional file 2). Low expression of one of them, *HLA-F*, was associated with a poor outcome (p = 0.027, log-rank test, HR = 0.55, CI.95 = 0.32–0.94) (Fig. 5f). *HLA-F* codes for a non-classical HLA molecule that forms a heterodimer with B2M [39] and, although its expression was detected in several cell types, it was mainly enriched in TME cells including CAFs, NK and T cells (Fig. 5g). Moreover, high expression of hsa-miR-1914-3p was observed in EVs derived from melanoma bearer and high relapse risk patients (LFC = 2.63 and 1.54, p = 0.004 and 0.045, respectively, Wilcox test, Fig. 5h) when compared to healthy individuals. High levels of mir-1914 were also associated with worse survival in lung adenocarcinoma (p = 0.046, log-rank test, HR = 1.41, CI.95 = 1.004–1.96, Additional file 2).

## Discussion

It has become clear that the tumor microenvironment composition is directly related to response to treatment, metastasis and patients’ survival. Several studies report the diversity of immune cell populations present in the tumor site and consider them as possible therapeutic targets [7, 40]. Due to the high cellular heterogeneity of tumors, different bioinformatics approaches have been developed to infer their composition based on molecular data. In this work, we applied two different deconvolution approaches to infer the TME composition of metastatic melanoma samples and identified tumor groups with different TME profiles impacting patients’ overall survival. We also correlated the TME composition with the coding and non-coding gene expression profiles to identify possible interactions between melanoma cells and other cell types that compose the TME. Importantly, the differences observed were not related to the main molecular alterations found in melanoma cells as no classical mutation (*BRAF*, *N/H/K-RAS*, or *NF1* mutations, nor triple wild-type) [36] was enriched in the above-mentioned groups.

The TME enrichment results identified by the two approaches used here were overall concordant, even though each tool was trained with distinct groups of cell types. This allowed us to identify two highly stable groups bearing very distinct TME compositions and prognostic values, G2 and G3. The G1 group is composed of regional lymph node metastasis samples, supporting the enriched B-cell signature observed, and presented an intermediate outcome. Samples from G2, of which the patients showed better prognosis compared to those from G3, were enriched in tumor-infiltrating lymphocytes (TILs), especially CD8+ T cells, reinforcing their canonical role and association with improved survival. G2 samples were also enriched in CD4+ T memory cells, which were previously demonstrated to, among other functions, boost antitumor response by supporting the persistence of CD8+ T cells [41].

We also identified a subset of patients (G3 group) whose TME presented a stronger M0 macrophages signature and worse clinical outcome when compared to G2. A similar TME profile and clinical outcome was observed in ER-positive breast tumors [42, 43] but was not previously described for melanoma. However, since the M0 macrophage phenotype [10, 44] has not been previously described in vivo, it could be an exclusive phenotype observed only in in vitro assays. For this reason, we hypothesized that this M0 macrophage signature is possibly related to the presence of tissue-resident macrophages or other immunosuppressive cellular populations not accounted in the deconvolution analysis, such as myeloid-derived suppressor cells (MDSC) or other regulatory macrophages, what could explain the worse overall survival in G3. Concordantly, the higher signature of suppressor cells observed in G3, which accounts for MDSC and Treg cells, support this hypothesis. Further studies are required to assess the accuracy of this M0 macrophage signature in vivo.

One way to reinforce the TME findings is to search for lymphocyte receptors (TCR and BCR) as they are exclusive of T and Natural Killer T cells or B cells, respectively. The TCR and BCR repertoires seem to agree with the relative frequencies of T and B cells found in each group, as shown by the higher TCR chain clone count in G1 and G2, and BCRs in G1. These repertoires correspond to the different lymphocyte subtypes and account for about 50% of the identified TME in those groups. However, one limitation of analyzing bulk sequencing data is that it is not possible to identify the lymphocyte subtypes (e.g., Treg, CD8+) expressing these TCRs. The lower diversity index found in the G2 samples, when compared to G1, could be associated to an ongoing tumor-specific response in G2 represented by the expanded T cell clones, which would increase the proportion of cells but not their diversity [45]. The higher representation of immunoglobulin in G1 corroborates the higher fraction of B cells found in the deconvolution analysis for this subgroup. The low BCR clonality found in metastatic tumor samples from G3 compared to G1, associated with an increased IgM, may suggest that B cells in this group are unable to perform class switch and acquire new effector functions. For more accurate characterization of TCR/BCR repertoires, deeper sequencing and longer reads than those available for the TCGA samples are required, limiting further receptor diversity analysis and clone count and impairing broader correlations with the TME deconvolution results.

It is well known that mutation and neoantigen burden are positively correlated to immunogenicity [17], however, although G2 and G3 show similar mutation and neoepitope burden and share the same mutational signature profile, they present different immunogenic profiles. Four different findings suggested immune evasion mechanisms in G3: [1] the stronger signature of checkpoint molecules which could induce T-cell exhaustion or compromise leukocyte activation and impair the supported anti-tumor immune response [46]; the suppression of antigen processing and presenting pathway, evidenced by [2] the higher dissociation constant (KD) of neoepitopes that diminishes antigen:HLA-I stability, which has been previously observed in other studies [47] and [3] the higher frequency of neoantigens in samples bearing mutations in the antigen processing and presenting pathway; [4] and downregulation of genes related to antigen presentation [17–19].

Altogether, our results point to an immunosuppressed group of tumors (G3) characterized by a less immunogenic landscape associated with a stronger signature of immunosuppressive molecules, enrichment of an M0 macrophage signature, depletion of other immune cells signatures and limited TCR and BCR repertoires. Despite that, it is important to notice that no difference in lymphocyte infiltration from histopathological annotations was found between G2 and G3, suggesting a suppressor phenotype rather than the absence of lymphocyte infiltration.

According to our findings, this immunosuppressive niche found in G3 is potentially regulated by miRNAs that can modulate the expression of important genes both at intracellular and intercellular levels through, in this last case, tumor-derived extracellular vesicles as mediators of cell–cell communication. One example that potentially contributes to the immunosuppressive phenotype found in G3 is the highly expressed mir-149, which has already been reported as dysregulated in many types of cancer including melanoma, where its upregulation influences the expression of both oncogenes and tumor suppressor genes [48]. In this study, we showed that the inhibition of its target, *NLRC5*, interferes with neo-antigen presentation, in agreement with other studies where it has been associated with immune evasion mechanisms and considered as a biomarker of immune surveillance [18].

Also favoring an immunosuppressive environment, we identified the circulating mir-1914 representing a putative player in the crosstalk between melanoma cells and the TME, regulating the expression of *HLA-F*. Although not directly involved in classical antigen presentation, *HLA-F* has been shown to cooperate in the cross presentation of activated lymphocytes [49] and to interact with inhibitory Killers Ig-like receptors (KIR) that are expressed by T and NK cells [50]. The downregulation of *HLA-F* in the cell surface of TME cells and the consequent decrease in the interaction with inhibitory KIR could increase the cytotoxic activity of NK towards CAF, T, and NK cells [50]. Based on that, we hypothesize that the release of mir-1914-containing EVs by melanoma cells could negatively modulate the expression of *HLA-F* in leukocytes and other TME cells and consequently increase NK cytotoxic activity towards TME cells, what could explain the higher percentage of necrotic cells in G3.

In contrast, high levels of the mir-150 was associated with good prognosis in three tumor types and, in G2 melanoma samples, it was highly correlated with the presence of CD8+ T cells. Consistent with our findings, this miRNA was previously reported to be highly expressed in B and T cells [51] and higher levels of mir-150 were found to inhibit proliferation, migration, and invasion of melanoma cells [52]. In accordance with our findings, mir-150 has also been associated with better patient’s survival [52] and suggested as a potential metastatic melanoma biomarker [53], while the expression of one of its target in malignant cells, *HILPDA*, was shown to be induced by hypoxia and associated with tumor resistance to anti-angiogenesis treatments [54]. Therefore, we hypothesize that mir-150 could be acting through lymphocyte-derived EVs to downregulate *HILPDA* expression in tumor cells. Similar to mir-150, we identified mir-342 as a potential marker of good prognosis in metastatic melanoma and breast carcinoma. This miRNA has already been reported as upregulated in metastatic melanomas compared to nevi and as a biomarker for post-recurrence survival in melanoma [55]. Concordantly, one of its potential targets, *PPM1F*, has been reported to promote migration and invasion in breast cancer cells [56]. This is in line with our observation that *PPM1F* is expressed by malignant cells in metastatic melanomas and its expression is associated with worse prognosis.

Others miRNAs with significant impact on patient survival across different tumor types were also found to be differentially expressed in the EV external dataset, further corroborating our findings of circulating miRNAs participating in the crosstalk between TME and malignant cells and supporting their putative role as prognostic biomarkers.

## Conclusions

Our results support the existence of a crosstalk between the tumor microenvironment and melanoma cells, where gene expression in the tumor is modulated by miRNAs from TME and vice versa. They also suggest a role for circulating miRNAs in driving immune evasion mechanisms. Taking together, the results presented here shed light on the role of the melanoma immune microenvironment in the progression and evolution of the disease. It also highlights the need to characterize the gene expression profile in the TME subpopulations at the cellular level to better understand their gene expression regulation mechanisms and how they may influence each aspect of the melanoma microenvironment. Finally, this knowledge is valuable for the diagnosis and evaluation of treatment response, since one can directly determine circulating tumor-derived molecules and whether a specific subpopulation has been ablated or altered by treatment.

## Supplementary information

**Additional file 1: Figure S1.** Tumor microenvironment of metastatic melanoma. **Figure S2.** Correlation between TME cells in metastatic melanoma samples. **Figure S3.** Mutational signatures and burden for each TME-associated group. **Figure S4.** Differentially expressed genes and miRNA. **Figure S5.** Kaplan–Meier curves of the differentially expressed mRNAs from MTG pairs. **Figure S6.** Survival estimates among the differentially expressed miRNAs from MTG pairs. **Figure S7.** Single-cell miRNA-target gene expression by cell type. **Figure S8.** Circulating miRNA expression levels found in extracellular vesicles from plasma samples. **Table S1.** Clinical-pathological data of the samples. **Table S2.** Genes considered in the antigen processing and presentation pathway. **Table S3.** Mutations identified in genes belonging to the antigen processing and presentation pathway. **Table S4.** Top 20 differentially expressed genes in G3 when compared to G2. **Table S5.** Top 20 differentially expressed miRNA in G3 when compared to G2.

**Additional file 2.** Negatively correlated MTG pairs. MTG pairs identified with a negative Pearson correlation coefficient of − 0.4 or less are listed. We also included information about the differential expression test, such as log fold change (LFC) and adjusted p-values (G3 related to G2) and about the survival log-rank test, such as hazard ratio (HR), confidence interval (CI) and p-value, for both target genes and miRNA that present impact on overall survival (High versus Low). We also indicate if the miRNAs were identified as circulating according to the miRandola database and putative cell of origin of the genes listed. MTG: miRNA-target gene. LFC: Log Fold Change. HR: Hazard Ratio. CI: Confidence Interval.

## Data Availability

The datasets supporting the conclusions of this article are available in the GEO repository (GEO accession GSE100508), https://www.ncbi.nlm.nih.gov/geo, dbGaP repository (accession phs000178), https://dbgap.ncbi.nlm.nih.gov/ and TCGA Research Network (accession SKML, LUAD, BRCA, ESCA, OV), https://www.cancer.gov/tcga. We also downloaded gene expression data from extracellular vesicles from Lee et al. [21].
