# Supplementary material for: Poor clinical outcome in metastatic melanoma is associated with a microRNA-modulated immunosuppressive tumor microenvironment
Source: J Transl Med. 2020 Feb 5;18:56. doi: 10.1186/s12967-020-02235-w (PMC7001250; doi:10.1186/s12967-020-02235-w)
Supplement: Supplementary file 1 — Additional file 1: Figure S1. Tumor microenvironment of metastatic melanoma. Figure S2. Correlation between TME cells in metastatic melanoma samples. Figure S3. Mutational signatures and burden for each TME-associated group. Figure S4. Differentially expressed genes and miRNA. Figure S5. Kaplan–Meier curves of the differentially expressed mRNAs from MTG pairs. Figure S6. Survival estimates among the differentially expressed miRNAs from MTG pairs. Figure S7. Single-cell miRNA-target gene expression by cell type. Figure S8. Circulating miRNA expression levels found in extracellular vesicles from plasma samples. Table S1. Clinical-pathological data of the samples. Table S2. Genes considered in the antigen processing and presentation pathway. Table S3. Mutations identified in genes belonging to the antigen processing and presentation pathway. Table S4. Top 20 differentially expressed genes in G3 when compared to G2. Table S5. Top 20 differentially expressed miRNA in G3 when compared to G2. [file 12967_2020_2235_MOESM1_ESM.docx]

**Additional file 1**

**Poor clinical outcome in metastatic melanoma is associated with a microRNA-modulated immunosuppressive tumor microenvironment**

Natasha Andressa Nogueira Jorge^1*^, Jéssica G. V. Cruz^1*^, Marco A. M. Pretti^1,2*^, Martin H. Bonamino^2,3^, Patricia A. Possik^2&^ e Mariana Boroni^1&^

*authors contributed equally to this work

**
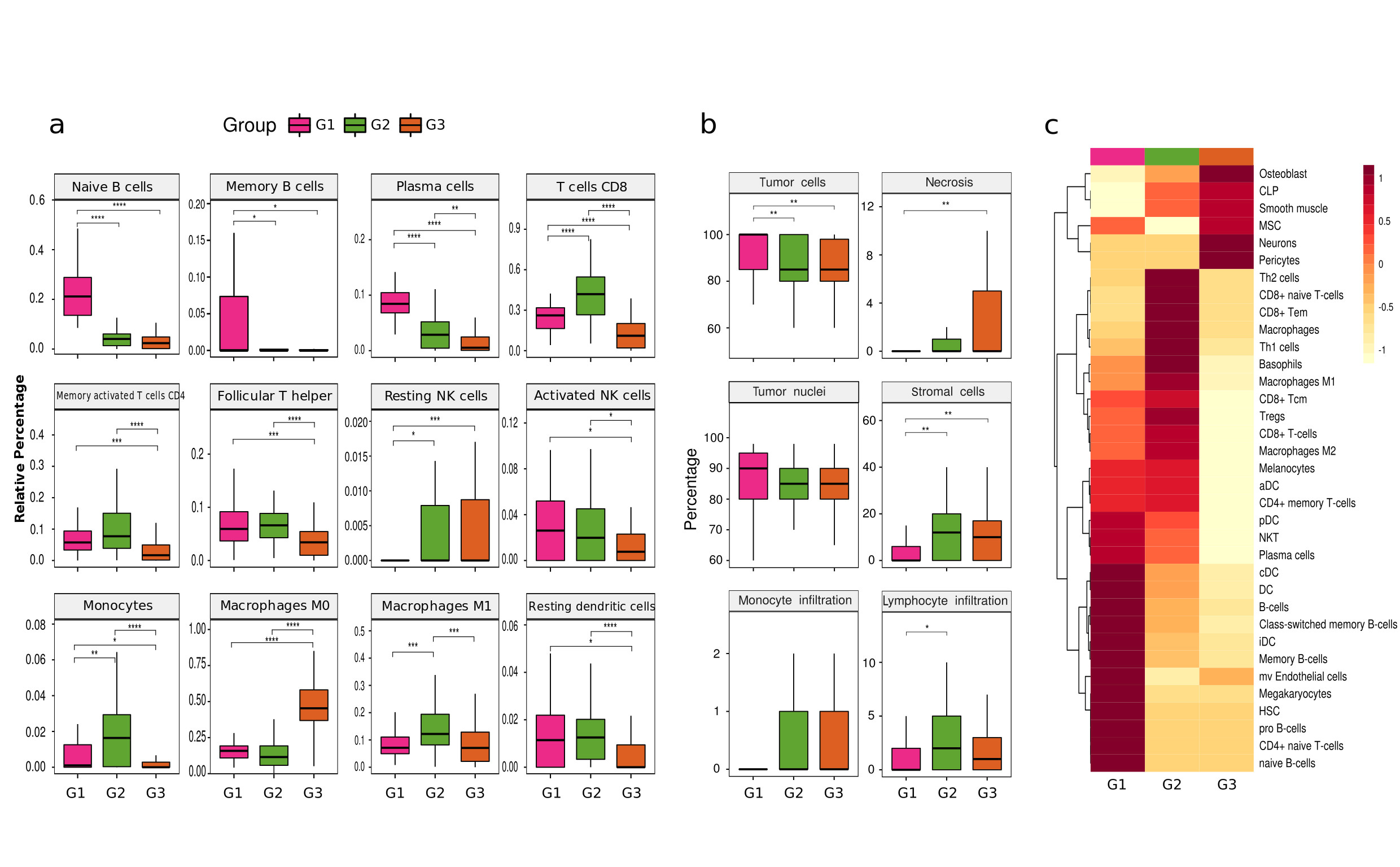
**

**Figure S1. Tumor microenvironment of metastatic melanoma.**

**a** Relative fractions of tumor, normal and stromal cells, tissue necrosis and leukocyte infiltration obtained from tissue slide annotations according to the three groups: G1 in pink, G2 in green and G3 in Orange. **b** Relative frequency of immune cell types per group predicted with cibersort. Only statistical significant results are shown. **c** Non-supervised clustering of median scores for immune and non-immune cell signatures performed by xCell for the three groups. Colors were attributed according to the z-score transformed scale. Horizontal label bars recapture assigned colors for each group. CLP = Common lymphocyte progenitor. MSC = Mesenchymal stem cell. Th = T helper. Tem = T effector memory. Tregs = T regulatory. aDC = activated dendritic cell. pDC = plasmacytoid dendritic cell. NKT = Natural killer T-cell. iDC = induced dendritic cell. Mv Endothelial cells = Microvascular endothelial cells. HSC = Hematopoietic stem cell. Mann-Whitney test was used for distribution comparisons. * p ≤ 0.05, ** p ≤ 0.01, ***: p <= 0.001 , ****: p <= 0.0001.

**
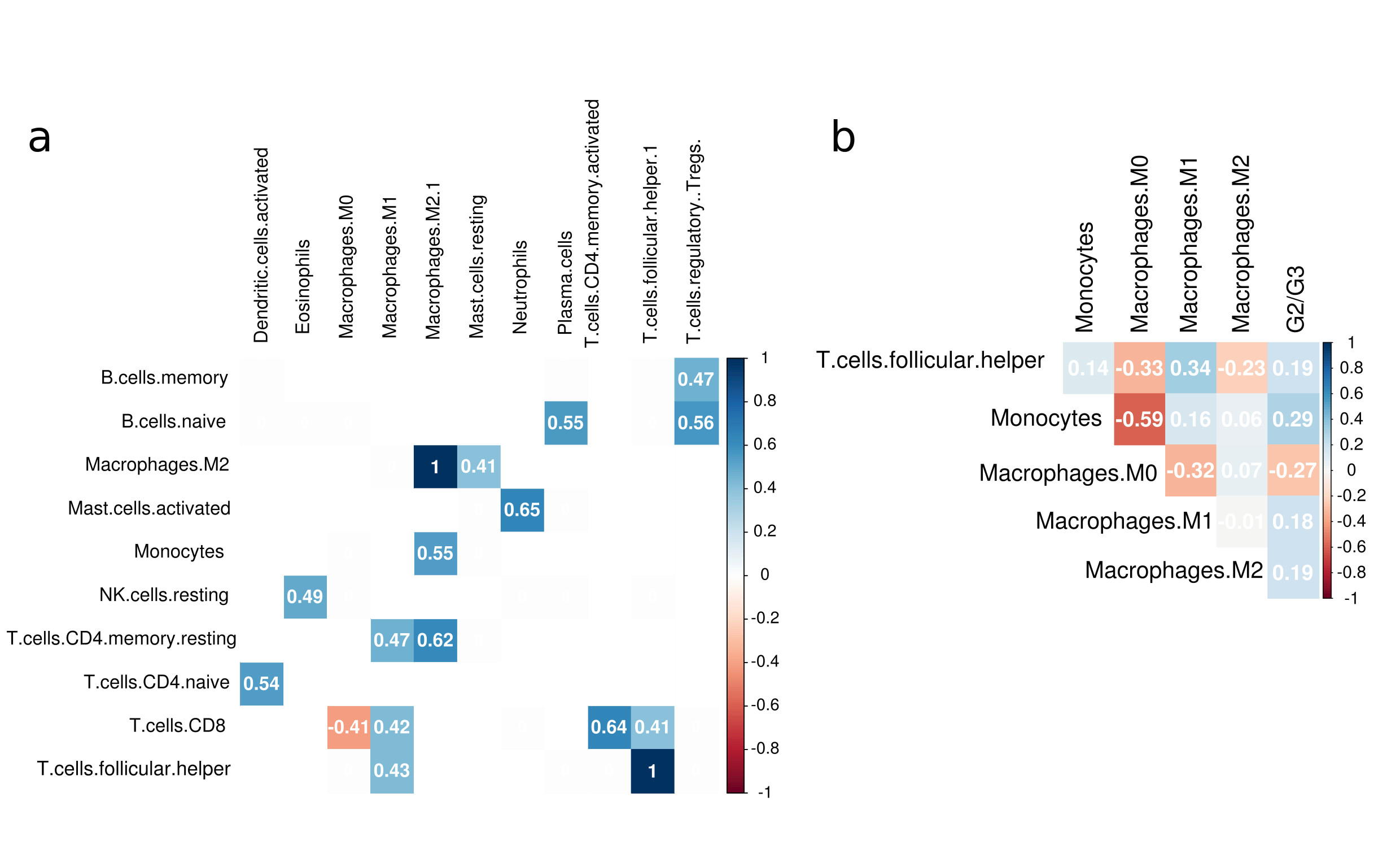
**

**Figure S2. Correlation between TME cells in metastatic melanoma samples.**

**a** Correlation matrix showing the Pearson correlation coefficient between relative frequency of immune cell types obtained from CIBERSORT considering the three groups together. Only statistical significant correlations are shown (p ≤ 0.05, abs(r) ≥ 0.4). Colors were scaled according to the coefficients. **b** Point biserial correlation matrix of immune cell types relative frequency to G2 and G3. Positive values in the last column denote positive correlation to G2 and negative values denote positive correlation to G3. Colors were scaled according to the Pearson correlation coefficients. Only statistical significant correlations are shown (p ≤ 0.05, abs(r) ≥ 0.15).

**
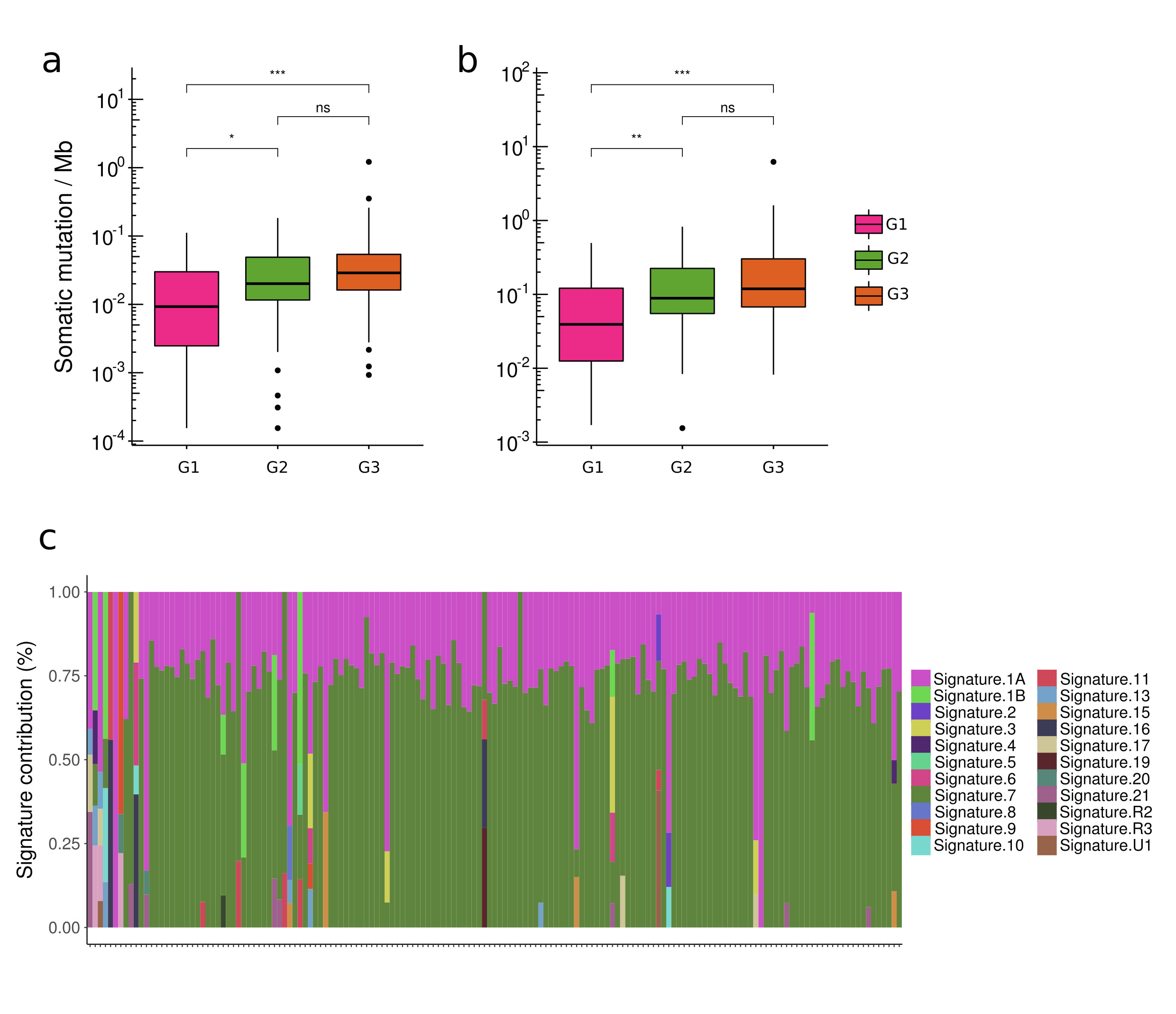
**

**Figure S3. Mutational signatures and burden for each group.**

Somatic mutation burden (in log) per megabase (Mb) depicted for **a** exonic and intronic **b** mutations for each group. Mann-Whitney’s test was used for distribution comparisons. *p < 0.05, **p < 0.01. **c** Mutational signature deconvolution per sample of each group using mutational signature.


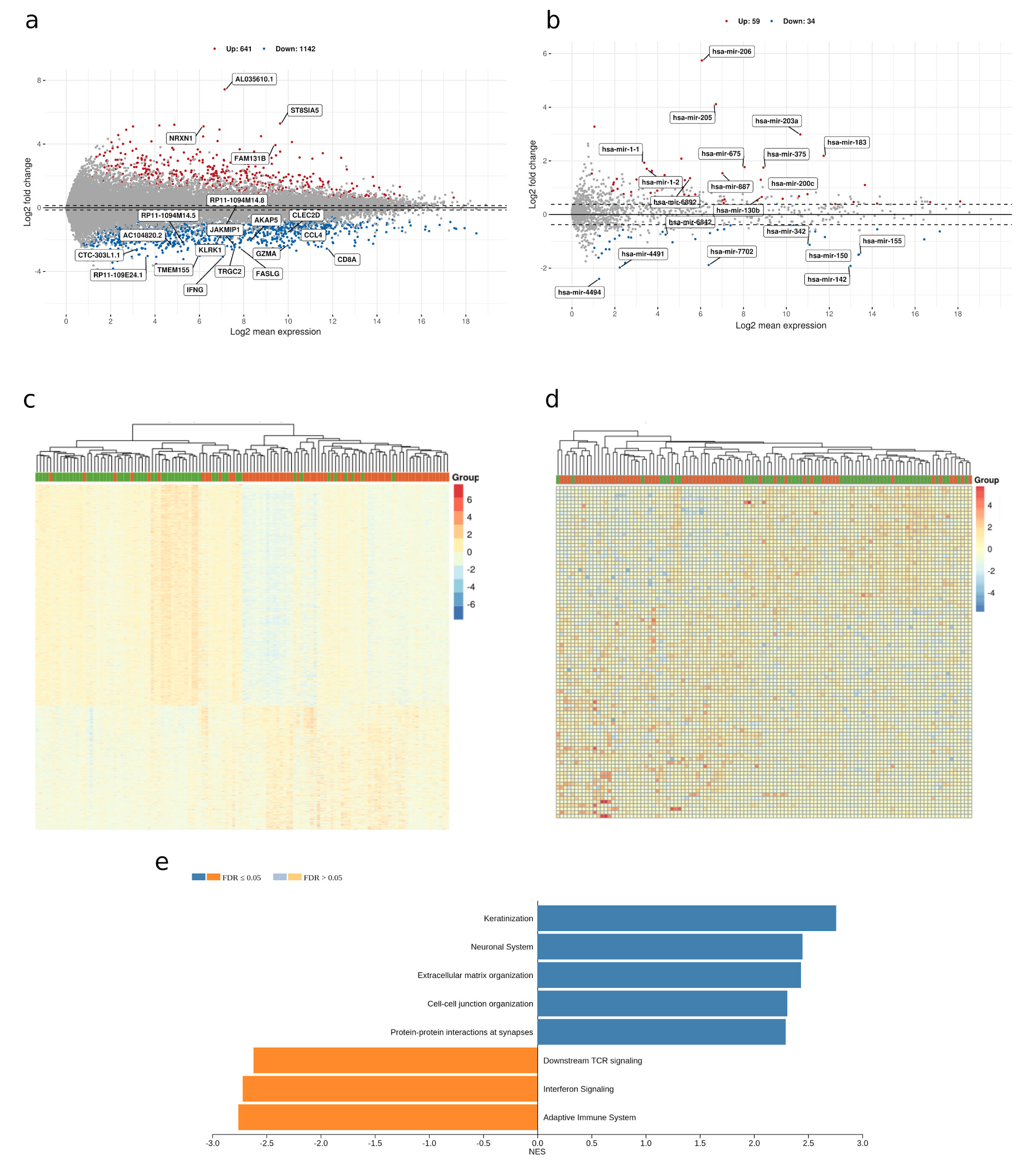


**Figure S4. Differentially expressed genes and miRNA.**

MAplot showing **a** up- and downregulated genes with an adjusted p-value ≤ 0.001, and **b** up- and downregulated miRNA with and adjusted p-value ≤ 0.05, in G3, when compared with G2. The top 20 DEG and miRNAs were labeled. Upregulated genes are shown in red and downregulated ones in blue. Heatmap of **c** DEG and **d** DEM, where samples were hierarchically clustered using euclidean distance. **e** GSEA of the differentially expressed genes using the REACTOME database. Pathways enriched in G3 are in blue and the ones enriched in G2 in orange. Only pathways with FDR ≤ 0.05 are shown.

**
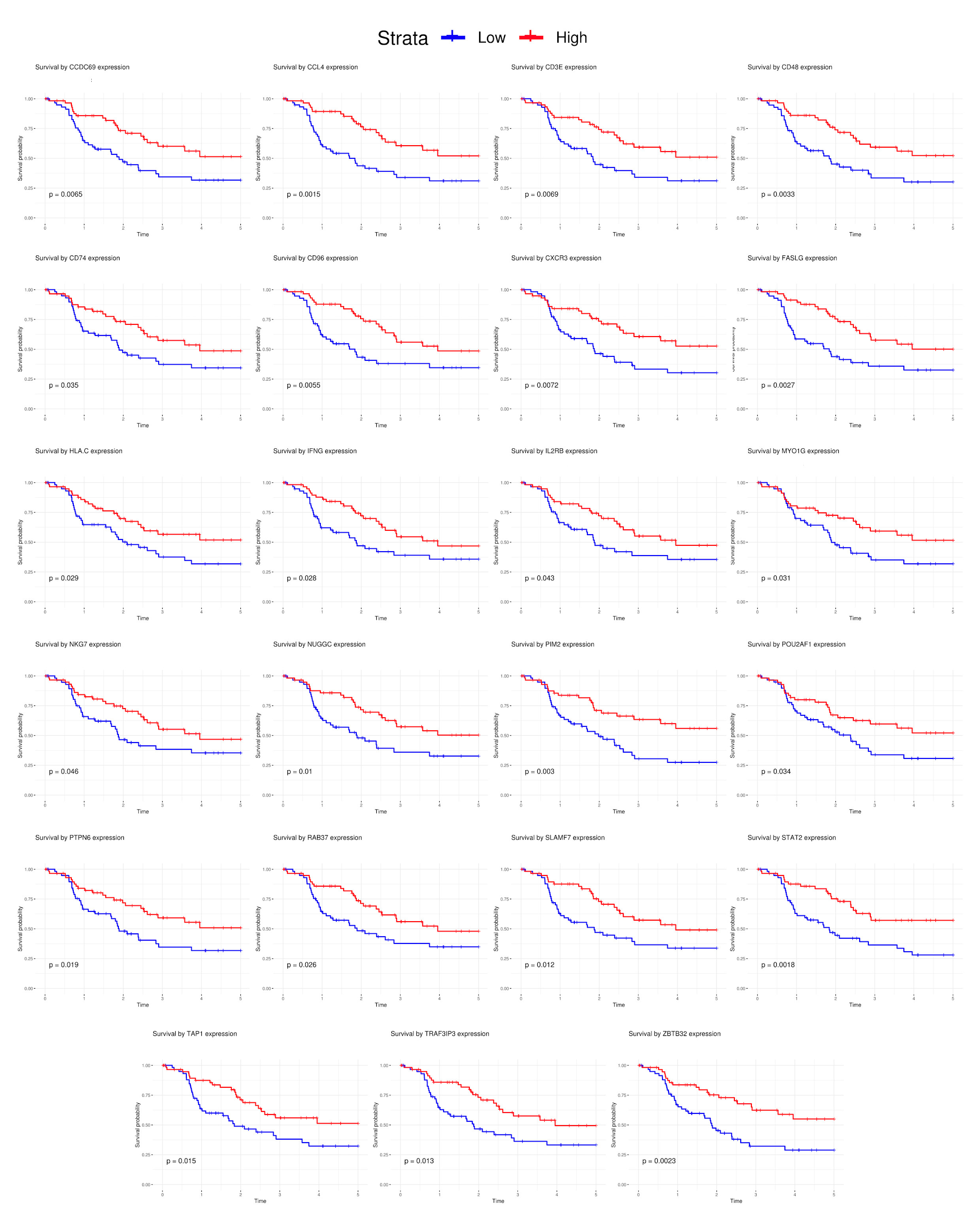
**

**Figure S5. Kaplan–Meier estimates of survival among the differentially expressed mRNAs from MTG pairs.**

Curves were based on patients from G2 and G3. Predicted target genes from miR-149, miR-150, miR-342 and miR-1914 showing significant impact on survival (p ≤ 0.05, log-rank test) are shown. For each gene, samples were classified as Low or High when expressing, respectively, lower or higher levels than the average gene expression.

**
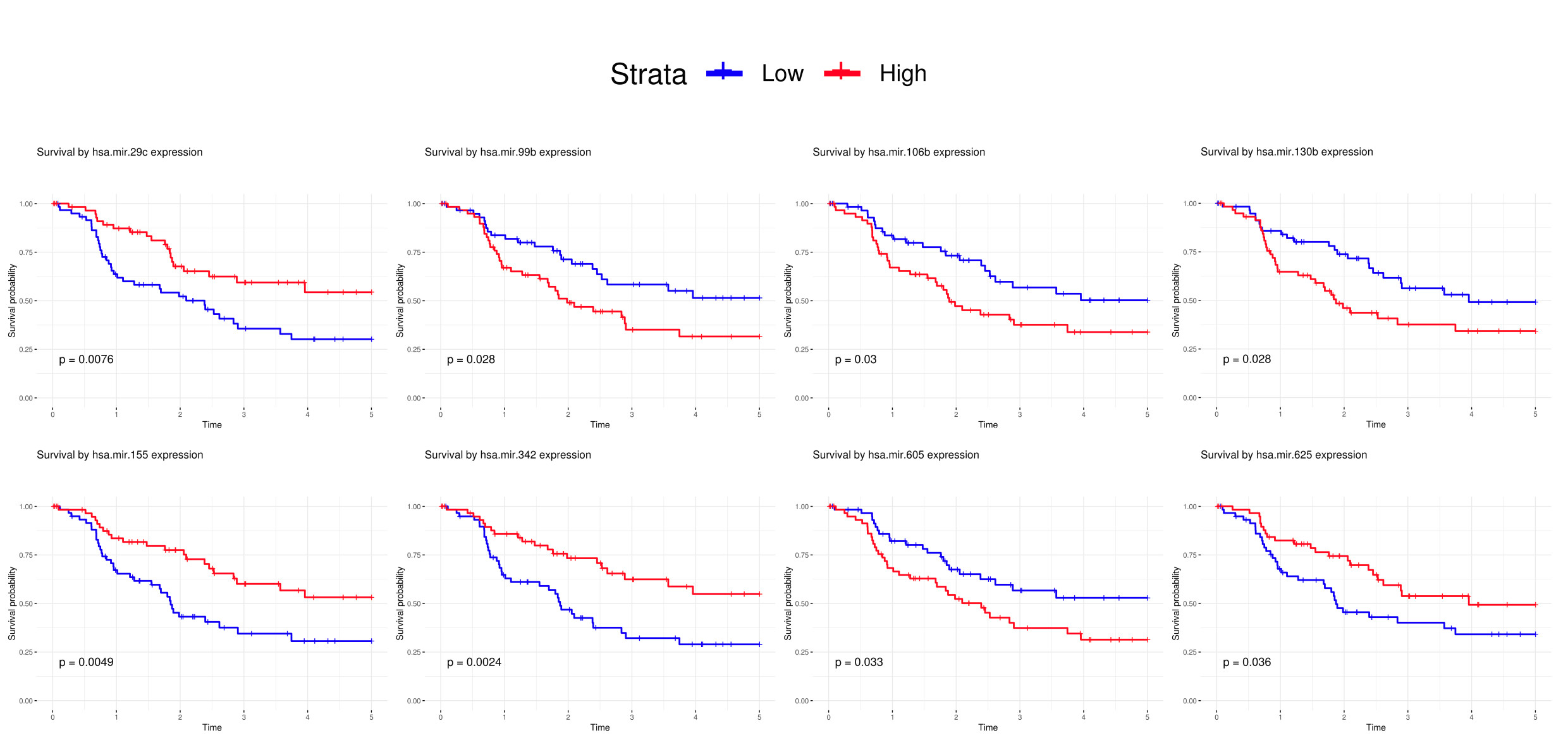
**

**Figure S6. Kaplan–Meier estimates of survival among the differentially expressed miRNAs from MTG pairs.**

Curves were based on patients from G2 and G3. Genes showing significant impact on survival (p ≤ 0.05, log-rank test) are shown. For each gene, samples were classified as Low or High when expressing, respectively, lower or higher levels than the average gene expression.

**
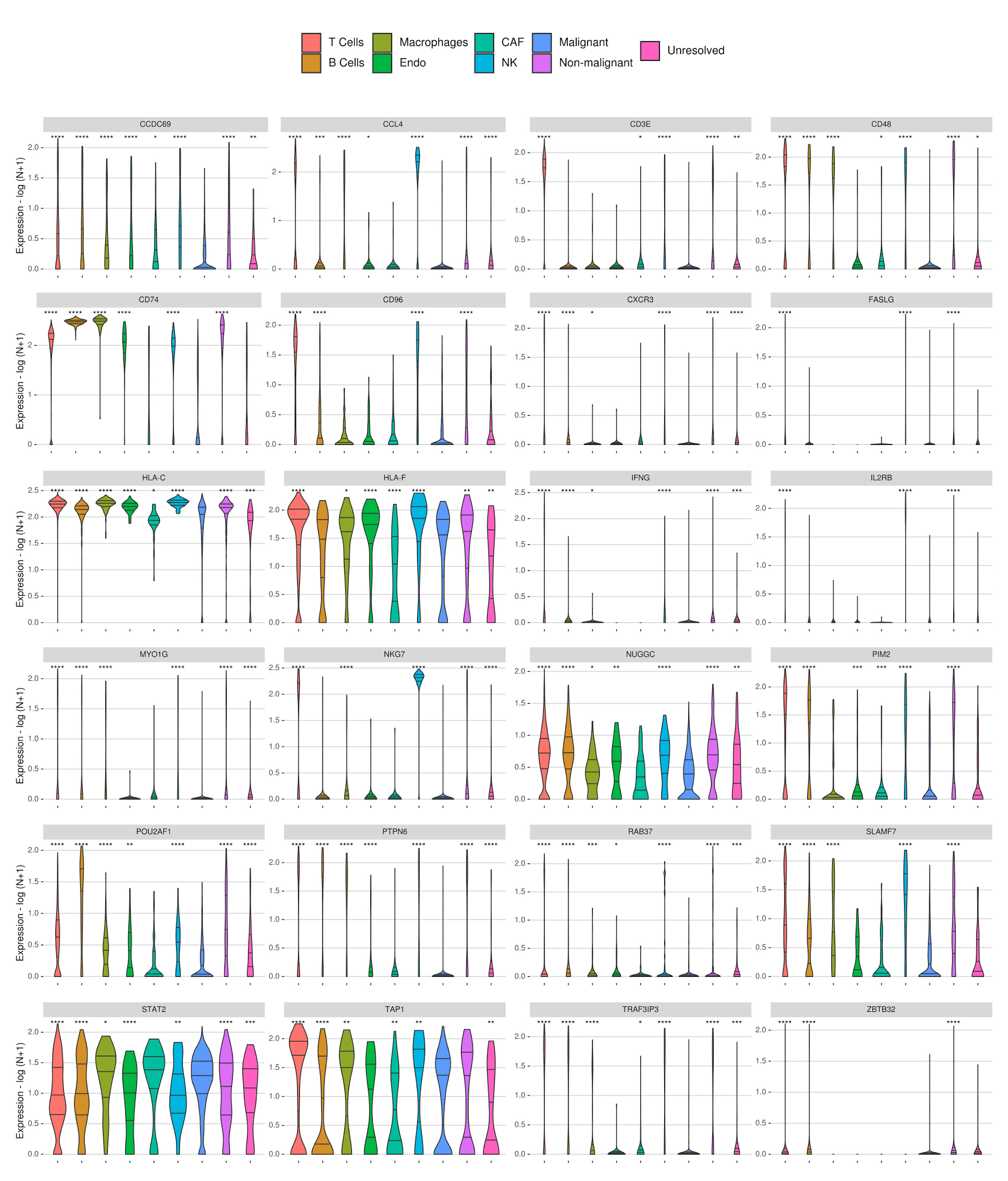
**

**Figure S7. Single-cell miRNA-target gene expression by cell type.**

[mRNA](https://www.sciencedirect.com/topics/neuroscience/messenger-rna) expression levels of potential miRNA-target genes among different cell populations are shown in violin plots. Asterisks inform on Mann-Whitney p values using the malignant cell type as the reference for the test. *: p <= 0.05, **: p <= 0.01, ***: p <= 0.001 , ****: p <= 0.0001.

**
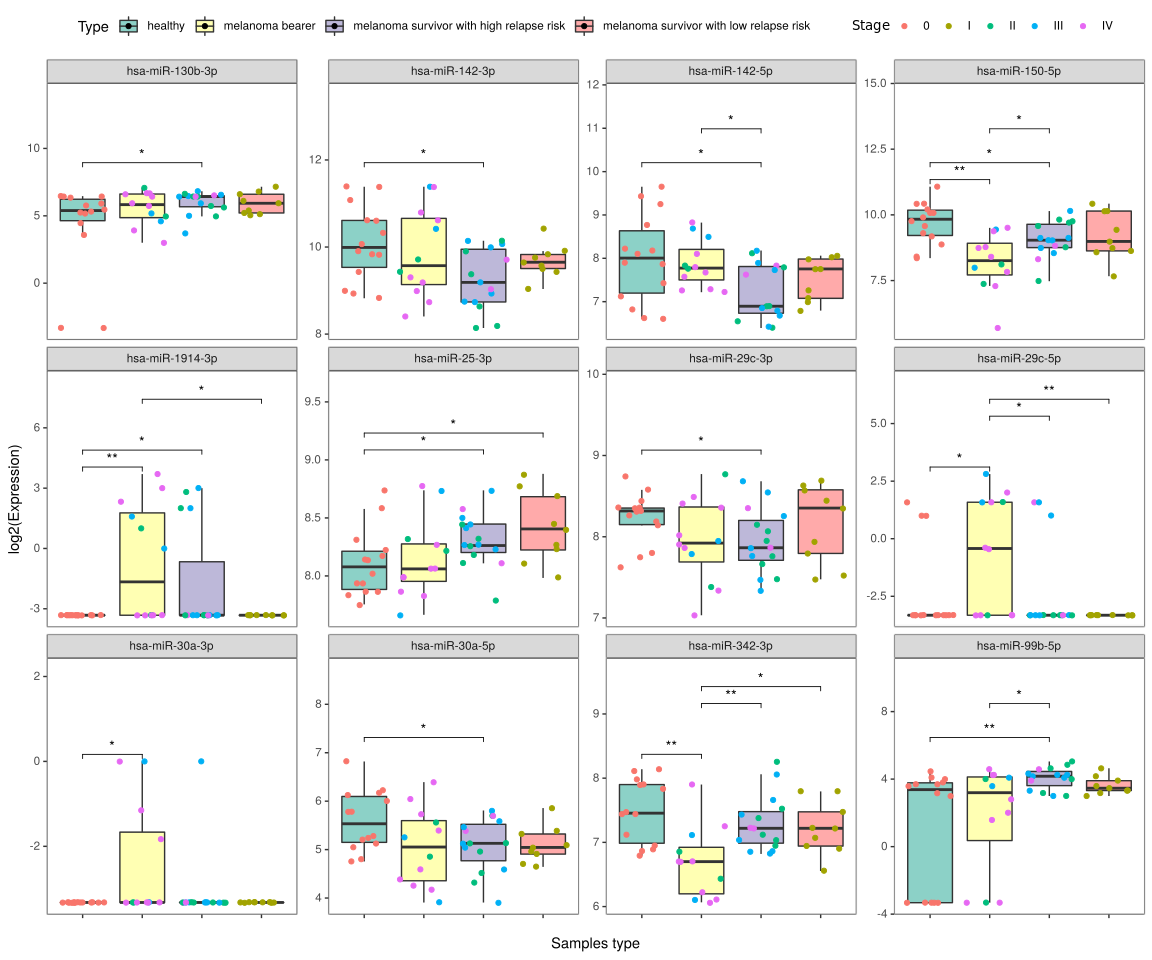
**

**Figure S8. Circulating miRNA expression levels found in extracellular vesicles from plasma samples.**

Groups were defined as healthy for control patients, melanoma barer for patients with metastatic melanoma tumors and melanoma survivor with high or low relapse risk for R0-operated patients with relapse relapse-risk based on tumor staging. Data is also colored according to tumor stage. Wilcox test was performed to identify significant differences in means between groups. Asterisks inform on Mann-Whitney p values. *: p <= 0.05, **: p <= 0.01, ***: p <= 0.001 , ****: p <= 0.0001.

**Table S1. Clinical-pathological data of the samples.** Information on clinical and pathological information on the samples for the whole cohort and each group. Pvalue for Fisher's exact test concerns the comparison of the three groups. Asterisks show variables that are enriched on that group for that feature according to residual values analysis, with * = p < 0.05 and ** = p < 0.01.

|  |  | **Whole Cohort (N=159)** | |  | **Group 1 (N=37)** | |  | **Group 2 (N=57)** | |  | **Group 3 (N=65)** | |  |  |
| --- | --- | --- | --- | --- | --- | --- | --- | --- | --- | --- | --- | --- | --- | --- |
| **A. Patient Demographic Data and Clinical Information** | | | | | | | | | | | | | | Fisher's exact test |
| Age (at diagnosis), in years |  | years | |  | years | |  | years | |  | years | |  | (p-value) |
| Mean (Range) |  | 57 (19-86) | |  | 59 (23-86) | |  | 56 (26-82) | |  | 58 (19-85) | |  |  |
| Median |  | 58 | |  | 60 | |  | 55 | |  | 60 | |  |  |
| Gender |  |  | % |  |  | % |  |  | % |  |  | % |  | 0.68600 |
| Male |  | 104 | 65.41 |  | 22 | 59.46 |  | 38 | 66.67 |  | 44 | 67.69 |  |  |
| Female |  | 55 | 34.59 |  | 15 | 40.54 |  | 19 | 33.33 |  | 21 | 32.31 |  |  |
| Ethnicity |  |  | % |  |  | % |  |  | % |  |  | % |  | 0.72200 |
| White |  | 151 | 94.97 |  | 35 | 94.59 |  | 54 | 94.74 |  | 62 | 95.38 |  |  |
| Asian |  | 3 | 1.89 |  | 1 | 2.70 |  | 1 | 1.75 |  | 1 | 1.54 |  |  |
| Black or African American |  | 1 | 0.63 |  | 1 | 2.70 |  | 0 | 0.00 |  | 0 | 0.00 |  |  |
| Not informed |  | 4 | 2.52 |  | 0 | 0.00 |  | 2 | 3.51 |  | 2 | 3.08 |  |  |
| **B. Clinical, Molecular and Pathological Features** | | | | | | | | | | | | | |  |
| Anatomic site of biospecimen |  |  | % |  |  | % |  |  | % |  |  | % |  | 1.43E-06 |
| Regional Lymph Node |  | 106 | 66.67 |  | 37 | 100.00 | ** | 36 | 63.16 |  | 33 | 50.77 |  |  |
| Regional Skin/Soft Tissue |  | 32 | 20.13 |  | 0 | 0.00 |  | 13 | 22.81 |  | 19 | 29.23 | * |  |
| Distant Metastasis |  | 21 | 13.21 |  | 0 | 0.00 |  | 8 | 14.04 |  | 13 | 20.00 | * |  |
| mRNA TCGA Subtypes |  |  | % |  |  | % |  |  | % |  |  | % |  | 2.51E-06 |
| Immune |  | 86 | 54.09 |  | 26 | 70.27 | * | 39 | 68.42 | * | 21 | 32.31 |  |  |
| Keratin |  | 10 | 6.29 |  | 1 | 2.70 |  | 2 | 3.51 |  | 7 | 10.77 |  |  |
| MITF-Low |  | 21 | 13.21 |  | 1 | 2.70 |  | 1 | 1.75 |  | 19 | 29.23 | ** |  |
| Not identified |  | 42 | 26.42 |  | 9 | 24.32 |  | 15 | 26.32 |  | 18 | 27.69 |  |  |
| Methylation TCGA Subtypes |  |  | % |  |  | % |  |  | % |  |  | % |  | 0.12000 |
| CpG island-methylated |  | 16 | 10.06 |  | 2 | 5.41 |  | 4 | 7.02 |  | 10 | 15.38 |  |  |
| Hyper-methylated |  | 31 | 19.50 |  | 7 | 18.92 |  | 10 | 17.54 |  | 14 | 21.54 |  |  |
| Hypo-methylated |  | 28 | 17.61 |  | 4 | 10.81 |  | 10 | 17.54 |  | 14 | 21.54 |  |  |
| Normal-like |  | 42 | 26.42 |  | 15 | 40.54 |  | 18 | 31.58 |  | 9 | 13.85 |  |  |
| Not identified |  | 42 | 26.42 |  | 9 | 24.32 |  | 15 | 26.32 |  | 18 | 27.69 |  |  |
| Mutation TCGA Subtypes |  |  | % |  |  | % |  |  | % |  |  | % |  | 0.40200 |
| Triple Wild-type |  | 37 | 23.27 |  | 12 | 32.43 |  | 11 | 19.30 |  | 14 | 21.54 |  |  |
| NF1 |  | 15 | 9.43 |  | 2 | 5.41 |  | 5 | 8.77 |  | 8 | 12.31 |  |  |
| RAS |  | 46 | 28.93 |  | 8 | 21.62 |  | 22 | 38.60 |  | 16 | 24.62 |  |  |
| BRAF |  | 61 | 38.36 |  | 15 | 40.54 |  | 19 | 33.33 |  | 27 | 41.54 |  |  |
| **C. Clinical Follow-up** | | | | | | | | | | | | | |  |
| Overall Survival, in years |  |  |  |  |  |  |  |  |  |  |  |  |  |  |
| Median (Range) |  | 1.85 (0-15.4) | |  | 1.88 (0-10.3) | |  | 2.11 (0-15.4) | |  | 1.25 (0-9.2) | |  |  |
| Vital Status at last follow-up |  |  | % |  |  | % |  |  | % |  |  | % |  | 0.07810 |
| Alive |  | 79 | 49.69 |  | 17 | 45.95 |  | 35 | 61.40 |  | 27 | 41.54 |  |  |
| Dead |  | 80 | 50.31 |  | 20 | 54.05 |  | 22 | 38.60 |  | 38 | 58.46 |  |  |
| Neoplasm cancer status |  |  | % |  |  | % |  |  | % |  |  | % |  | 0.01980 |
| Tumor Free |  | 67 | 42.14 |  | 15 | 40.54 |  | 33 | 57.89 | ** | 19 | 29.23 |  |  |
| With Tumor |  | 86 | 54.09 |  | 21 | 56.76 |  | 22 | 38.60 |  | 43 | 66.15 | * |  |
| Not informed |  | 6 | 3.77 |  | 1 | 2.70 |  | 2 | 3.51 |  | 3 | 4.62 |  |  |

**Table S2. Genes considered in the antigen processing and presentation pathway.** The gene names and respective ENSEMBL IDs were obtained from the antigen processing and presentation pathway of Kyoto Encyclopedia of Genes and Genomes (KEGG) database considering the genes that participate in MHC-I antigen presentation.

| **ENSEMBL** | **Gene** |
| --- | --- |
| [ENSG00000166710](http://www.ensembl.org/Homo_sapiens/geneview?gene=ENSG00000166710) | *B2M* |
| [ENSG00000179218](http://www.ensembl.org/Homo_sapiens/geneview?gene=ENSG00000179218) | *CALR* |
| [ENSG00000127022](http://www.ensembl.org/Homo_sapiens/geneview?gene=ENSG00000127022) | *CANX* |
| [ENSG00000164307](http://www.ensembl.org/Homo_sapiens/geneview?gene=ENSG00000164307) | *ERAP1* |
| [ENSG00000164308](http://www.ensembl.org/Homo_sapiens/geneview?gene=ENSG00000164308) | *ERAP2* |
| [ENSG00000044574](http://www.ensembl.org/Homo_sapiens/geneview?gene=ENSG00000044574) | *HSPA5* |
| [ENSG00000092010](http://www.ensembl.org/Homo_sapiens/geneview?gene=ENSG00000092010) | *PSME1* |
| [ENSG00000100911](http://www.ensembl.org/Homo_sapiens/geneview?gene=ENSG00000100911) | *PSME2* |
| [ENSG00000131467](http://www.ensembl.org/Homo_sapiens/geneview?gene=ENSG00000131467) | *PSME3* |
| [ENSG00000167004](http://www.ensembl.org/Homo_sapiens/geneview?gene=ENSG00000167004) | *PDIA3* |
| [ENSG00000168394](http://www.ensembl.org/Homo_sapiens/geneview?gene=ENSG00000168394) | *TAP1* |
| [ENSG00000204267](http://www.ensembl.org/Homo_sapiens/geneview?gene=ENSG00000204267) | *TAP2* |
| [ENSG00000231925](http://www.ensembl.org/Homo_sapiens/geneview?gene=ENSG00000231925) | *TAPBP* |

**Table S3. Mutations identified in genes belonging to the antigen processing and presentation pathway.** For each mutation in genes from the Supplementary Table 2 identified across samples are shown the reference (REF) and altered (ALT) allele(s); gene name; predicted impact and protein consequence (Conseq.) from the Variant Effect Predictor; genomic coordinate based on GRCh38 (Coord.); and variant ID from dbSNP or Catalogue Of Somatic Mutations In Cancer (COSMIC) databases. NA. Information not available. MOD. Moderate

| **Sample** | **REF** | **ALT** | **Gene** | **Impact** | **Conseq.** | **Group** | **chr** | **Coord.** | **Variant ID** |
| --- | --- | --- | --- | --- | --- | --- | --- | --- | --- |
| TCGA-D3-A8GD | ACT | A | *B2M* | HIGH | frameshift | 1 | 15 | 44711582 | NA |
| TCGA-EE-A2MD | ACTCT | A | *B2M* | HIGH | frameshift | 1 | 15 | 44711582 | NA |
| TCGA-EE-A2MS | A | AC | *ERAP2* | HIGH | frameshift | 2 | 5 | 96889350 | NA |
| TCGA-ER-A195 | C | T | *TAP2* | MOD | missense | 2 | 6 | 32830737 | rs745691531 |
| TCGA-D3-A1Q4 | A | T | *HSPA5* | MOD | missense | 3 | 9 | 125238172 | rs1449162533 COSM3654334 |
| TCGA-D3-A3MU | G | A | *ERAP1* | MOD | missense | 3 | 5 | 96797302 | COSM3618891 |
| TCGA-D3-A8GM | G | A | *PSME2* | MOD | missense | 3 | 14 | 24145434 | NA |
| TCGA-DA-A1HY | G | A | *TAP2* | HIGH | stop_gained | 3 | 6 | 32835676 | COSM4605827 |
| TCGA-FR-A7U9 | C | T | *CALR* | MOD | missense | 3 | 19 | 12940283 | rs1353439888 |
| TCGA-FW-A3R5 | G | A | *TAP2* | MOD | missense | 3 | 6 | 32830698 | rs1469773653 COSM4620485 |
| TCGA-W3-AA1V | C | T | *CANX* | HIGH | stop_gained | 3 | 5 | 179720558 | NA |
| TCGA-W3-AA1V | GC | G | *PSME3* | HIGH | frameshift | 3 | 17 | 42841545 | rs1269425860 |
| TCGA-Z2-AA3V | G | A | *ERAP2* | MOD | missense | 3 | 5 | 96903463 | rs1229133455 |

**Table S4. Top 20 differentially expressed genes in G3 when compared to G2.**

| **Genes** | **log2FoldChange** | **padj** |
| --- | --- | --- |
| lincRNA AL035610.1 | 7.43 | 2.69E-30 |
| ST8 Alpha-N-Acetyl-Neuraminide Alpha-2,8-Sialyltransferase 5 (ST8SIA5) | 5.29 | 1.24E-20 |
| Neurexin 1 (NRXN1) | 5.11 | 6.64E-20 |
| Interferon Gamma (IFNG) | -3.08 | 9.18E-20 |
| Transmembrane Protein 155 (TMEM155) | -3.01 | 5.98E-18 |
| lincRNA RP11-109E24.1 | -3.03 | 8.04E-18 |
| Family With Sequence Similarity 131 Member B (FAM131B) | 3.93 | 1.17E-17 |
| T-Cell Surface Glycoprotein CD8 Alpha Chain (CD8A) | -2.57 | 2.65E-17 |
| Killer Cell Lectin Like Receptor K1 (KLRK1) | -2.44 | 2.65E-17 |
| lncRNA AC104820.2 | -2.36 | 2.73E-17 |
| Fas Ligand (FASLG) | -2.49 | 2.80E-17 |
| C-C Motif Chemokine Ligand 4 (CCL4) | -2.14 | 4.86E-17 |
| Granzyme A (GZMA) | -2.37 | 6.06E-17 |
| lincRNA RP11-1094M14.8 | -2.22 | 9.93E-17 |
| A-Kinase Anchoring Protein 5 (AKAP5) | -2.05 | 1.16E-16 |
| lncRNA RP11-1094M14.5 | -2.29 | 1.55E-16 |
| C-Type Lectin Domain Family 2 Member D (CLEC2D) | -1.63 | 1.63E-16 |
| T Cell Receptor Gamma Constant 2 (TRGC2) | -2.47 | 1.87E-16 |
| CTC-303L1.1 | -2.61 | 1.98E-16 |
| Janus Kinase And Microtubule Interacting Protein 1 (JAKMIP1) | -2.40 | 3.84E-16 |

**Table S5. Top 20 differentially expressed miRNA in G3 when compared to G2.**

| **Genes** | **log2FoldChange** | **padj** |
| --- | --- | --- |
| mir-206 | 5.74 | 1.20E-10 |
| mir-142 | -1.92 | 2.24E-10 |
| mir-203a | 2.99 | 1.38E-09 |
| mir-183 | 2.19 | 2.98E-08 |
| mir-205 | 4.11 | 4.72E-08 |
| mir-7702 | -1.88 | 5.73E-07 |
| mir-342 | -1.13 | 6.53E-07 |
| mir-4494 | -2.40 | 1.71E-06 |
| mir-155 | -1.49 | 1.71E-06 |
| mir-4491 | -1.97 | 1.91E-06 |
| mir-6892 | 1.35 | 7.29E-06 |
| mir-150 | -1.50 | 1.23E-05 |
| mir-675 | 1.77 | 5.73E-05 |
| mir-887 | 1.54 | 7.17E-05 |
| mir-200c | 1.29 | 2.64E-04 |
| mir-375 | 1.75 | 2.64E-04 |
| mir-1-1 | 1.93 | 4.74E-04 |
| mir-1-2 | 1.70 | 7.92E-04 |
| mir-6842 | -0.75 | 8.44E-04 |
| mir-130b | 0.65 | 1.75E-03 |
